# An Intelligent Individualized Cardiovascular App for Risk Elimination (iCARE) for Individuals With Coronary Heart Disease: Development and Usability Testing Analysis

**DOI:** 10.2196/26439

**Published:** 2021-12-13

**Authors:** Yuling Chen, Meihua Ji, Ying Wu, Qingyu Wang, Ying Deng, Yong Liu, Fangqin Wu, Mingxuan Liu, Yiqiang Guo, Ziyuan Fu, Xiaoying Zheng

**Affiliations:** 1 School of Nursing, Capital Medical University Beijing China; 2 Advanced Innovation Center for Human Brain Protection, Capital Medical University Beijing China; 3 Along Technology Inc Beijing China; 4 The Asia-Pacific Economic Cooperation Health Science Academy, Peking University Beijing China; 5 Institute of Population Research, Peking University Beijing China

**Keywords:** mobile health, health behavior, system, development, usability, coronary heart disease

## Abstract

**Background:**

Death and disability from coronary heart disease (CHD) can be largely reduced by improving risk factor management. However, adhering to evidence-based recommendations is challenging and requires interventions at the level of the patient, provider, and health system.

**Objective:**

The aim of this study was to develop an Intelligent Individualized Cardiovascular App for Risk Elimination (iCARE) to facilitate adherence to health behaviors and preventive medications, and to test the usability of iCARE.

**Methods:**

We developed iCARE based on a user-centered design approach, which included 4 phases: (1) function design, (2) iterative design, (3) expert inspections and walkthroughs of the prototypes, and (4) usability testing with end users. The usability testing of iCARE included 2 stages: stage I, which included a task analysis and a usability evaluation (January to March 2019) of the iCARE patient app using the modified Health Information Technology Usability Survey (Health-ITUES); and stage II (June 2020), which used the Health-ITUES among end users who used the app for 6 months. The end users were individuals with a confirmed diagnosis of CHD from 2 university-affiliated hospitals in Beijing, China.

**Results:**

iCARE consists of a patient app, a care provider app, and a cloud platform. It has a set of algorithms that trigger tailored feedback and can send individualized interventions based on data from initial assessment and health monitoring via manual entry or wearable devices. For stage I usability testing, 88 hospitalized patients (72% [63/88] male; mean age 60 [SD 9.9] years) with CHD were included in the study. The mean score of the usability testing was 90.1 (interquartile range 83.3-99.0). Among enrolled participants, 90% (79/88) were satisfied with iCARE; 94% (83/88) and 82% (72/88) reported that iCARE was useful and easy to use, respectively. For stage II usability testing, 61 individuals with CHD (85% [52/61] male; mean age 53 [SD 8.2] years) who were from an intervention arm and used iCARE for at least six months were included. The mean total score on usability testing based on the questionnaire was 89.0 (interquartile distance: 77.0-99.5). Among enrolled participants, 89% (54/61) were satisfied with the use of iCARE, 93% (57/61) perceived it as useful, and 70% (43/61) as easy to use.

**Conclusions:**

This study developed an intelligent, individualized, evidence-based, and theory-driven app (iCARE) to improve patients’ adherence to health behaviors and medication management. iCARE was identified to be highly acceptable, useful, and easy to use among individuals with a diagnosis of CHD.

**Trial Registration:**

Chinese Clinical Trial Registry ChiCTR-INR-16010242; https://tinyurl.com/2p8bkrew

## Introduction

Coronary heart disease (CHD) is the leading cause of cardiovascular death, accounting for 7.3 million annual deaths worldwide [1,2], with about 130,000 being reported from China alone [3]. Preventive interventions focusing on unhealthy behavior (diet, physical activity, smoking) modification and adherence to secondary prevention medications can reduce at least 47% of CHD mortality and decrease 68% of CHD-related major risk factors [3-7]. Although interventions facilitating behavior change and medication adherence have been developed and widely implemented, the prevalence of unhealthy behaviors and medication nonadherence remained high among individuals with CHD, including those who have already experienced life-threatening cardiac events or underwent percutaneous coronary interventions [6,8-11]. In large international studies from Europe and China, such as the EUROASPIRE IV and the Improving Care for Cardiovascular Disease in China-Acute Coronary Syndrome (CCC-ACS) Project, over half of patients with CHD had substantial unhealthy behaviors (50%-77%), with a large proportion of patients not adhering to prescribed preventive medications (45%-83%) [8,10,12-16].

Effective intervention targeting adherence to healthy behaviors and medications requires strategies to be implemented in addressing multiple CHD-related risk factors [17]. In addition, as reported by previous studies, evidence-based interventions with incorporation of real-time monitoring, person-centered care, and tailored feedback are essential to ensure affordable and sustainable long-term benefits [17-21]. However, it is difficult for conventional interventions to provide real-time monitoring; therefore, these interventions are unable to deliver tailored feedback and person-centered care [13-15].

With the advancement in information and communication technologies, mobile health (mHealth)–based health management systems and apps have emerged [18-20]. These have shown high potential in providing individualized intervention and sending instant automatic feedback based on real-time monitoring, therefore it might have promising effects in impacting health behavior change and promoting medication adherence [18-20]. Currently, many mHealth apps are designed to promote physical activity [21], track diet intake [22], assist with smoking cessation [23], remind patients to take medications [24,25], and facilitate self-management of CHD risk factors [26]. However, many of the existing mHealth apps are not individualized; additionally, they are not inclusive of suggested strategies and often have limited functions on tailored feedback, especially automatic feedback, and inadequate information updates [27]. None of the top 5 downloaded mHealth apps in China incorporated multiple key health behaviors identified by clinical guidelines [27]. A recent systematic review on health behavior changes related to physical activity, diet, drug and alcohol use, and mental health revealed that the majority of the mHealth apps (40/52, 80%) for health behavior change only focused on a single behavior, with the other 20% focusing on 2 health behaviors [18]. Meanwhile, studies suggested that currently available mHealth apps, including those in the management of CHD, failed to address users’ needs and preferences, or consider their unique characteristics during the app development phase [23-25]. Furthermore, health care providers, such as nurses, are often not involved in the design and development of the mHealth apps, despite them being recognized as important for designing interventions that are reflective of patients care needs [27,28]. In addition, currently available mHealth apps were often not guided by a behavior change theory in developing their interventions [24,29], and they often do not sufficiently emphasize evidence-based interventions [28].

In light of the imperative needs for health behavior modification and medication management among individuals with CHD, we developed an Intelligent Individualized Cardiovascular App for Risk Elimination (iCARE) through facilitating healthy behavior and medication adherence. iCARE was designed to address the gaps in conventional interventions, in which multiple CHD-related risk factors were managed through real-time monitoring, person-centered care, and automatic tailored feedback. Following clinical guidelines on CHD secondary prevention [3,30,31], the interventions of iCARE were developed based on the Intervention Mapping framework [32], and the Contemplation–Action–Maintenance (CAM) model [33,34], which was an integrated behavior change model describing the roles of multiple moderators and mediators during the motivation and volition stages that are essential for healthy behavior change and behavior maintenance in individuals with CHD (Multimedia Appendix 1). In our preliminary study, we generated a set of iCARE interventions along with a set of “IF–THEN” algorithms to improve patients’ adherence to health behaviors and medications, and conducted a needs assessment on the use of an mHealth-based system among patients with CHD [33]. The interventions developed in that preliminary work were incorporated as a built-in intervention bank into the cloud platform of the iCARE system during the development stage. The findings from the needs assessment were used to guide the design of the user interface of the iCARE system.

The purposes of this study were to describe the development of iCARE and to evaluate its usability among individuals with CHD. The effect of iCARE on facilitating adherence to actual behavior change is out of scope of this study, and it will be reported separately.

## Methods

### Development Process of iCARE

#### Overview

End users’ needs analysis and the development of interventions within iCARE have been published elsewhere and are summarized as preliminary work (Multimedia Appendix 1) [33]. Based on our preliminary work, we followed the user-centered design principle [20] in developing iCARE, and considered this principle as the main methodology for designing person-centered care–delivering systems [35]. The development process of iCARE consisted of 4 phases: (1) function design, (2) iterative design, (3) expert inspections and walkthroughs of prototypes, and (4) usability testing with end users. We established a multidisciplinary team including health care professionals (nurses specialized in cardiovascular care, nursing informaticians, nursing researchers), project managers, software engineers, software architects, and interface designers to develop iCARE. Weekly meetings were held to discuss the issues raised and advance the progress of system development among multidisciplinary team members throughout all phases.

#### Phase 1: Functional Design of iCARE

iCARE consisted of 3 functional components: a patient app for individuals with CHD and their families, a care provider app for health care providers such as nurses and physicians, and a cloud platform. The key functions and modules of each component were first drafted in a mind mapping software (XMind version 8; XMind, Ltd.) based on the results of the needs assessment and the overall aims of the system. Through brainstorming activities within the professional team, a detailed contextual document that described the framework, modules of each app and the cloud platform, functions, and design principles were formulated to guide the development of iCARE. To ensure the interventions are more effective and reflective of the care management of individuals with CHD, the components and schematic diagram of iCARE were designed following the nursing process [35].

#### Phase 2: Iterative Design of iCARE

##### Overview of Stages

In this phase, the system architecture, database, protective measures to secure individuals’ personal identification information and health data, and user interface were determined. The patient app and care provider app were developed to be compatible with Android-based smartphones, as they are more popular and affordable in China [27]. We will also develop iCARE to be compatible on iOS-based smartphones once the app is identified as being stable in Android-based smartphones.

##### Design Architecture of iCARE

To increase the scalability and ensure the reliability of the software, developers need to respond to user’s needs and consider continuous delivery to establish a culturally and environmentally adaptive software [36]. Therefore, the MicroService architecture [36-38], an emerging architectural design, was applied in the development of iCARE. MicroService is used for handling complex systems that require highly repetitive and frequent changes and allows continuous delivery of software in short circles; it was identified with increased deployability and modifiability among researchers. Compared with the monolithic approach, the MicroService architecture allows us to organize iCARE into a set of small, single-responsibility units, and self-contained services that can be developed, operated, tested, and deployed independently [36]. The MicroService architecture uses a continuous delivery approach to handle data queries, which makes it easier to achieve zero-downtime releases in responding to the rapid evolving needs [35]. After the MicroService architecture is applied, a majority of the services were allowed to use NoSQL databases to capture, query, and administer data in iCARE. This greatly reduced the time and effort in database migration, thereby substantially improving the efficiency in system development.

iCARE involves transmission of sensitive data, such as personal identification information, which makes the system subject to external and internal threats. Therefore, to ensure system security, protective measures were applied within iCARE. Based on the MicroService architecture, access control was performed through the User Account and Authentication service. The 2-way authentication HTTPS was applied to ensure the security of data transmission between the app and the back end services. To ensure secured data transmission and storage, all sensitive data in the apps were encrypted, and access to the data was password protected with sophisticated protective mechanisms, including verifying password strength, limiting number of login errors, periodically changing password, etc. Meanwhile, the system has built-in functions in providing database backup and recovery. In this study, we utilized MongoDB, a document-based database, and one of the leading NoSQL databases. MongoDB is used in the MicroService architecture owing to its ability to provide flexible schema, redundancy, automation, and scalability. The security of the MongoDB database is mainly achieved by strengthening the security of the operating system, authentication, and database. The development of iCARE was based on international and national data standards (Table S1 in Multimedia Appendix 1).

##### User Interface Design and Visualization Design

To fully reflect end users’ needs and achieve the overall objectives of iCARE, the health professional team initially drafted the user interfaces of the patient app using Axure RP 8 software (Axure Software Solutions, Inc.). First, the user interface of the patient app was designed with diet, physical activity, smoking, and medication adherence as the main functional structure. Second, as the interventions of iCARE were developed based on the CAM behavior change model, the user interface of the patient app was designed to consider the characteristics of the interventions that addressed the major moderators and mediators identified within the CAM model, with patients’ risk perception, outcome expectation, action planning, self-efficacy, social support, perceived enjoyment, perceived effectiveness, and coping planning addressed within the app designing process.

Based on the initial draft, the interface designer prepared a mock-up (draft) of the planned user interfaces and their interactions and workflow using Flinto version 26.0.5 [39] following the 6 user-friendly design principles: structure, simplicity, visibility, feedback, tolerance, and reuse [40]. To ensure workflow efficiency, prevent information-entry errors, and increase end users’ positive experience in using the app, contrasting color, large font, distinctive graphical shapes, etc. were utilized to indicate different functions and status, as well as to increase the accessibility and effectiveness of the app. Meanwhile, visualization (infographics, figures, and chart) was used to enhance individuals’ perception on the risks of nonadherence to health behavior, prescribed medications, etc.

#### Phase 3: Expert Inspections and Walkthroughs of the iCARE Prototypes

iCARE was programed using Java and Nodejs, with Linux and Docker as the operating environment. Following user-centered design principles, and based on the functions, content, and architecture of iCARE, the software engineers developed the fully functional prototypes (alpha and beta versions) of the patient app and care-provider app [41].

Following the agile approach [42], software engineers iteratively identified and solved technical/implementational issues during the development process. The alpha version was initially developed, released, tested, and retested among technology professionals. The Gitlab and Docker tools were implemented for the release management throughout different stages and environments. Meanwhile, to ensure testability of iCARE, we used the test-first mindset and practices to define the acceptance criteria for the system [36].

After resolving technical issues or bugs in the alpha version, a full version of the app, the beta version, was formulated. Multiple testing cycles of the beta version of the app were applied among health care professionals in our team until a final consensus was reached. The testing of the beta version lasted for 3 months before the fully functional beta version was found to be stable and ready for usability testing.

#### Phase 4: Usability Testing of iCARE With End Users

##### Overview of Stages

The usability testing of iCARE included 2 stages. Stage I included 2 steps: a task analysis and a usability evaluation of the patient app using the modified Health Information Technology Usability Survey (Health-ITUES) [43]. Stage II included a usability evaluation using the Health-ITUES among end users who used the app for 6 months. The usability testing was designed following the International Organization for Standardization (ISO) standard 9241-11 [44]. In this study, we only tested the usability of the patient app among individuals with CHD. As iCARE was in the testing stage, and the main focus of this study was to test the experience of patients and the usability of the patient app, and therefore, we did not include health care providers (such as nurses) to complete the Health-ITUES.

Eligible patients who were hospitalized in 3 cardiac units of 2 university-affiliated hospitals in Beijing, China, participated in stage I usability testing. Patients who had a documented diagnosis of CHD and reported at least one unhealthy behavior were included in this study. A more detailed description of study participants and procedures is provided in Multimedia Appendix 1. The study was approved by the Institutional Review Committee of the Capital Medical University (Approval No. 2015SY45) and the study hospitals (Beijing An-Zhen Hospital, Approval No. 2015030; Beijing Chao-Yang Hospital, Approval No. 20211224). All enrolled participants provided written informed consent. Fully functional beta version 1 was used for task analysis and first-step usability testing with the Health-ITUES; beta version 2 was used for stage II usability testing with the same questionnaire. As the usability study is the preliminary work of our randomized controlled trial (RCT), which tests the effects of iCARE, the entire study has been registered with the Chinese Clinical Trial Registry (No. ChiCTR-INR-16010242).

##### Task Analysis

To identify usability problems as suggested by Maramba et al [45], individuals with CHD were asked to complete 8 tasks regarding the main functions of the app (Table S2 in Multimedia Appendix 1). Eight videos on how to complete the tasks were made available to all participants (Multimedia Appendices 2-9). All participants watched each video and practiced the exercises on the patient app until they were fully confident in completing each task. Based on the ISO standard 9241-11 [44], using standardized evaluation forms, we described the usability of the patient app in terms of the duration in completing each task, the level of task completeness, usability error, and usability problems. The duration in completing each task was recorded using a timer. The level of task completeness (Table S3 in Multimedia Appendix 1) was ranked on a scale of 1 (no problem) to 4 (significant problem) [44]. The completeness rates for each task were calculated by the proportion of participants who successfully completed the tasks (scored as “1”). Any usability-related problems were identified when patients were not able to complete the task. The severity of usability problems (Table S4 in Multimedia Appendix 1) was ranked from 0 “I don’t agree that this is a usability problem at all” to 4 “usability catastrophes: imperative to fix this before product can be released,” to reflect the level of severity of the identified problems [46,47]. The usability problem rates for each task were reflected by the proportion of participants who reported any problems when completing the tasks. Based on the ISO standard 9241-11 [44], the effectiveness of the app refers to the level of task completeness by the users; the efficacy of the app is expressed by the time (in seconds) the end user required in completing the tasks.

##### Usability Evaluation Using the Questionnaire

The usability evaluation was completed using the modified Health-ITUES [43]. Stage I usability evaluation was conducted among participants who completed the task analysis on beta version 1 (January 2019 to March 2019). All participants were given a small gift (around US $4) after they completed the questionnaire to compensate for their time. The iCARE system was then refined according to the results of stage I usability testing, and the updated version was used in the RCT. Stage II usability evaluation was performed in June 2020 among individuals who enrolled in the RCT after they used the patient app for 6 months. The RCT was a multicenter open-labeled study that tested the effects of iCARE interventions on major cardiovascular risk reduction and facilitation of adherence to health behaviors and medication among individuals with CHD. The RCT has 3 groups, including the intervention group (received fully functional iCARE to provide person-centered interventions that use multiple formats such as comics, videos, pictures, words to address all the factors in the CAM model plus routine care), control group 1 (received a person-centered intervention presented in text format only but did not address all factors in the CAM model plus routine care), and control group 2 (received routine care). Participants who were randomized into the intervention arm in the RCT and used the app for at least six months were invited to complete the modified Health-ITUES. Details of the RCT protocol and the results of the trial will be published in the future. We also retrieved the number of times patients accessed the app and analyzed the patients’ preference by identifying the most commonly used functions of the app over the past 6 months.

### Measures

The English version of the Health-ITUES has a Cronbach α of .85-.92 and a criterion validity of 0.46-0.70 [43]. To evaluate the usability of the patient app, the questionnaire was customized to address the type of tool (patient app), the user (individuals with CHD), and tasks (for reducing cardiovascular risk factors) of the target app. The Chinese version of the Health-ITUES in evaluating the patient app was shown to be reliable and valid among individuals with CHD in this study, with the Cronbach α at .74-.90 and expert validity at 0.87-0.95. The Health-ITUES comprises 4 dimensions with 20 items: impact (3 items), perceived usefulness (9 items), perceived ease of use (5 items), and user control (3 items). Each item was rated from “1” (strongly disagree) to “5” (strongly agree) on a 5-point Likert scale. Total scores ranged from 20 to 100, with higher scores indicating better usability. In addition, iCARE was considered satisfactory, useful, and easy to use if the score of patients’ responses was 4 or more on item 7 (using iCARE is useful for self-management of CHD-related risk factors), item 9 (I am satisfied with iCARE for self-management of CHD-related risk factors), and item 14 (learning to operate iCARE is easy for me), respectively.

### Data Analysis

Statistical analysis was conducted using SPSS version 24.0 software (IBM, Corp). Categorical data were described as frequencies and proportions. Continuous data were tested for normality using the one-sample Kolmogorov–Smirnov test, and was described as means and SDs, or medians and interquartile ranges, as appropriate. Comparisons of participants’ responses to the Health-ITUES between the first and second usability testing groups, and comparisons between the usage in terms of different groups (working status, educational levels, gender, used the device or not) were performed with the Mann–Whitney U test (scores of the Health-ITUES) and chi-square test (rates of satisfaction, usefulness, and easy to use), as appropriate. A *P* value <.05 was considered statistically significant. Regarding the sample size for usability testing, according to a previous study [43], the average score for each item in the Health-ITUES is 4 points and the maximum SD for each item is 0.8. Based on this information, considering 75% of the enrolled participants could complete the questionnaire and following a 2-sided 1-sample *t* test (unpaired), we estimated that we would need a total of 77 individuals in the first usability evaluation to achieve 80% power with α=.05, with an expected average score of 4.3 on each item of the Health-ITUES. As the second usability testing was conducted among individuals who were enrolled in the ongoing RCT, we did not calculate the sample size for that test.

## Results

### Phase 1: Functional Design of iCARE

**Figure 1:**
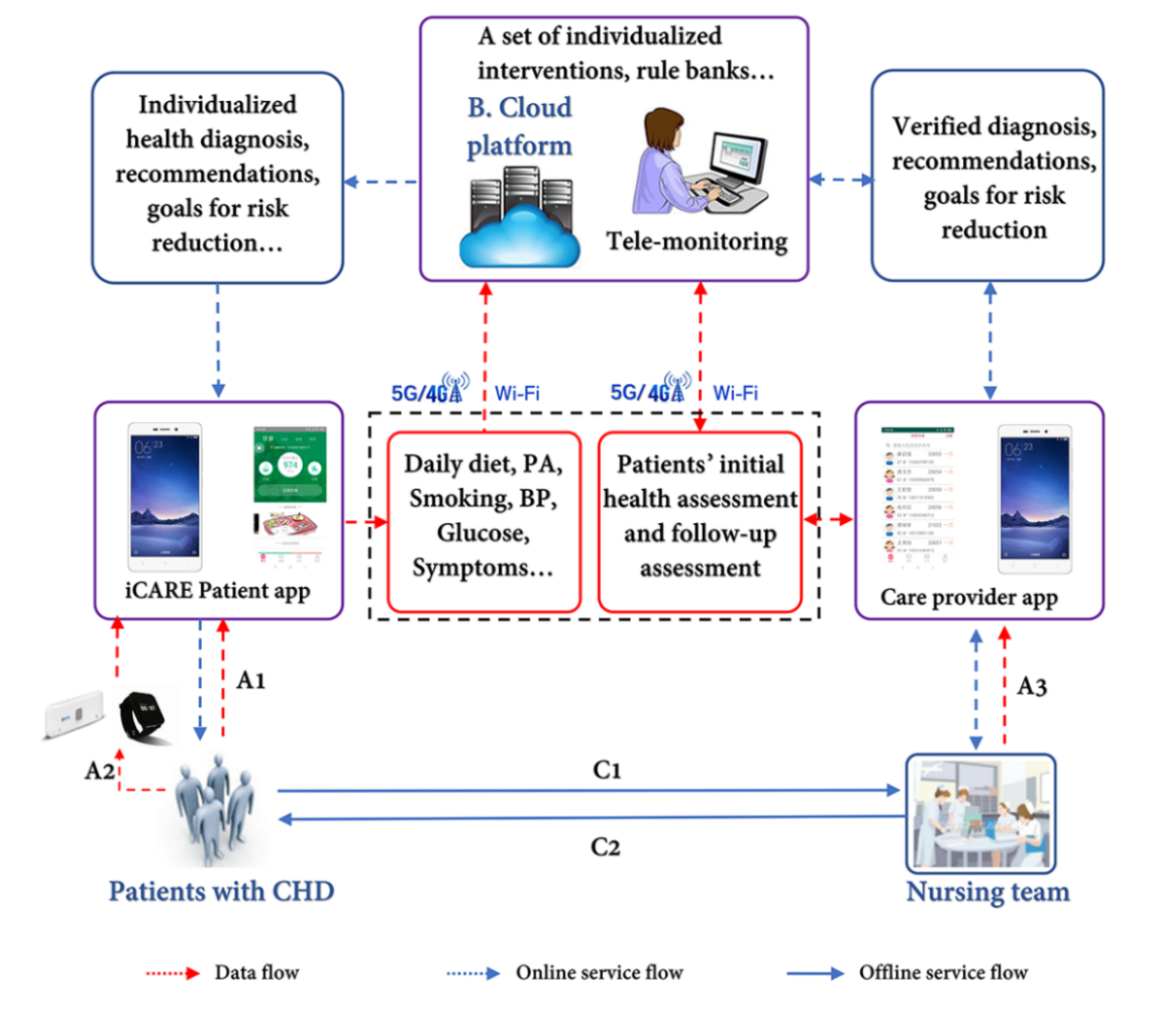
The components and schematic diagram of the iCARE. (A1) Initial and periodic assessment through daily monitoring via manual entry by patients. (A2) Daily monitoring by wearable devices. (A3) Patients’ initial assessment data, follow-up data. (C1) Initial and periodic assessment by cardiovascular nurses. (C2) Further evaluation, interventions, follow-up. iCARE: Intelligent Individualized Cardiovascular Application for Risk Elimination; PA: physical activity; BP: blood pressure; CHD: coronary heart disease.

The functional components of iCARE illustrating the system workflow are shown in Figure 1.

An initial health assessment was conducted by a nurse in our research team before patients were discharged from the hospital. The assessment included patients’ demographics (age, gender, education, etc.), current health behaviors (daily diet, physical activity, smoking status), preferences regarding health behaviors (such as preferred fruits, vegetables, types of physical activities), and medication adherence in the past 30 days before admission if patients were on medication. Patients’ health information including blood pressure, glucose level, lipid level, etc. was also collected. After the initial assessment, a summarized health report that described patient’s medical and nursing diagnosis, unhealthy behaviors, and modifiable risk factors, along with established goals for risk reduction, was generated for patient’s easy access through the patient app. The tailored goals for risk reduction were created based on the recommendations in the cardiovascular secondary prevention guidelines, with consideration of patients’ age, gender, preferences (such as preferred time and types of physical activity), physical activity levels, left ventricular ejection fraction, comorbidity, metabolic status, musculoskeletal condition or disease, cardiac risk profile, and the current level of habitual physical activity. When the health care providers completed the baseline assessments on the care provider app and uploaded the data to the cloud platform, the recommended final goals for cardiovascular risk reduction were generated based on the cardiovascular secondary prevention guidelines and patients’ specific health issues. They were then displayed in patients’ health report and on the home page of the patient app. Patients can personally modify their goals to be achieved at different stages if they felt the goals sent to the patient app were not achievable at the moment. When we designed the algorithm for the goals of healthy diet, we considered patients’ gender, age, and physical activity levels to determine the requirement of daily energy intake. Health-related data on physical activity, diet, blood pressure, lipid profile, blood sugar, heart rate, and weight were entered by patients either manually or through wearable devices. A smart watch (Ustone) was used to count steps and patients’ heart rate, and a sensor (*Youyi Tang*; only for patients with diabetes) was connected to iCARE to record blood glucose, and the information was uploaded to the cloud platform for analysis after patients logged into the app and selected the corresponding wearable device, which is to be synchronized, by clicking the upload button. Patients can also upload a photocopy of their laboratory results via the patient app if they prefer. Researchers will check the uploaded information in the care provider app or access it via cloud platform, and manually edit and send tailored feedback based on the laboratory results. A reminder on daily health data entry was sent to patients if they failed to enter data for 3 days, and a call from nurses was made if no further action was detected within 4 days after the reminder was sent. Instant and individualized feedback and tailored recommendations for changing behavior were automatically sent to the care provider app for verification by cardiovascular nurses based on the built-in algorithms (examples are shown in Multimedia Appendix 1). The verified recommendations of interventions were sent to the patient app for implementation by patients. Patient’s health behaviors and medication adherence were evaluated through daily monitoring and periodic assessment (every 3 months).

The overall functional modules of iCARE are displayed in Figure S1 in Multimedia Appendix 1. The patient app was developed with functions allowing individual end users to input personal data related to health behaviors, prescribed medications, and physiological indicators, and to review individual health data and health report, as well as to receive recommended interventions. The care provider app was developed with the functions allowing health care providers to view patients’ health data and health report, make health assessment, verify recommended interventions, provide health consultation as necessary, and manage follow-up visits. The cloud platform was developed for health care providers and system manager with authorized access rights to view patients’ overall health behaviors, prescribed medications, and physiological indicators. It was also structured to create and edit the intervention pool, knowledge, rule, and algorism; assign roles of users; and conduct data analysis. However, having access to the cloud platform and care provider app does not allow nurses or physicians to modify the data entered by patients.

### Phase 2: Iterative Design of iCARE

#### Architecture Design

**Figure 2:**
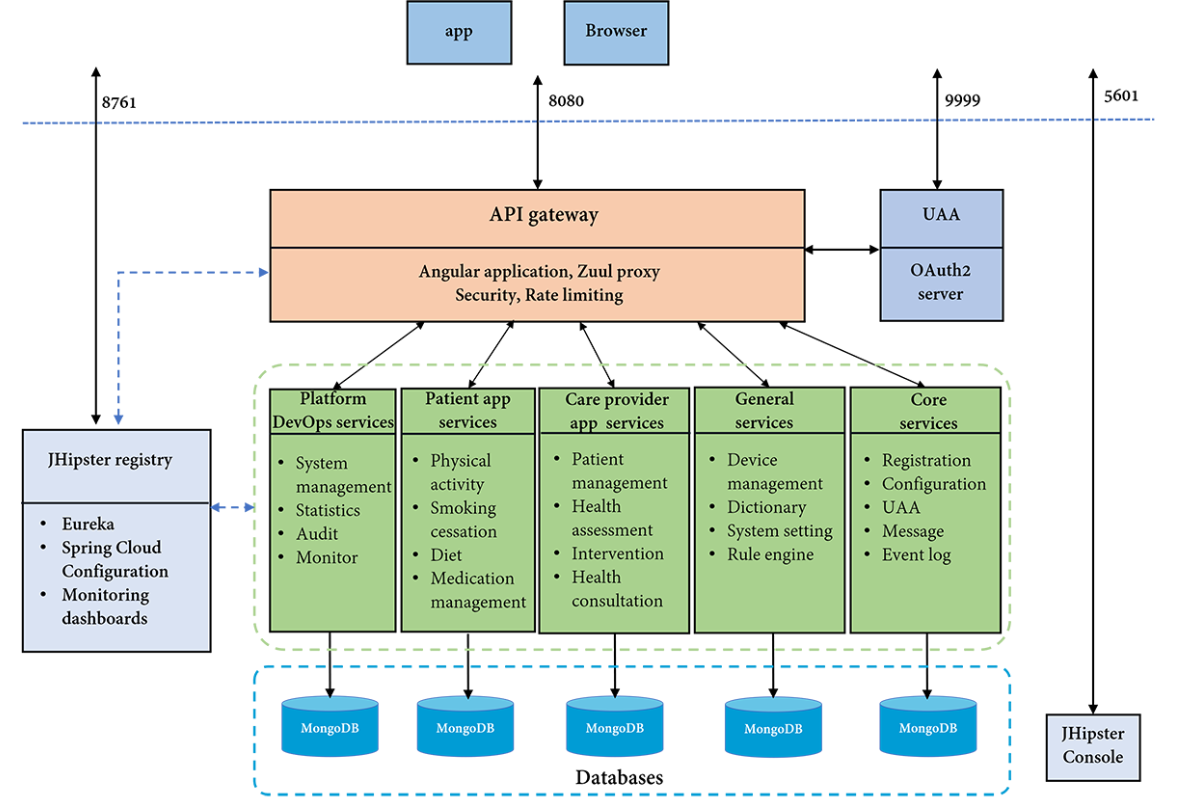
The system architecture of the iCARE (Intelligent Individualized Cardiovascular Application for Risk Elimination). API: Application Programming Interface; UAA: User Account and Authentication.

The MicroService architecture of iCARE is displayed in Figure 2. It included a set of microservices, such as services for the platform DevOps, patient app, and care provider app, and general and core services. Each individual microservice has its separate database (MongoDB) with its own domain data. Instead of calling services directly, the app and browser get access to the different services through the application programming interface gateway which will forward the request to the appropriate services on the back end. All services were connected with the JHipster registry.

#### User Interface Design and Visualization Design

**Figure 3:**
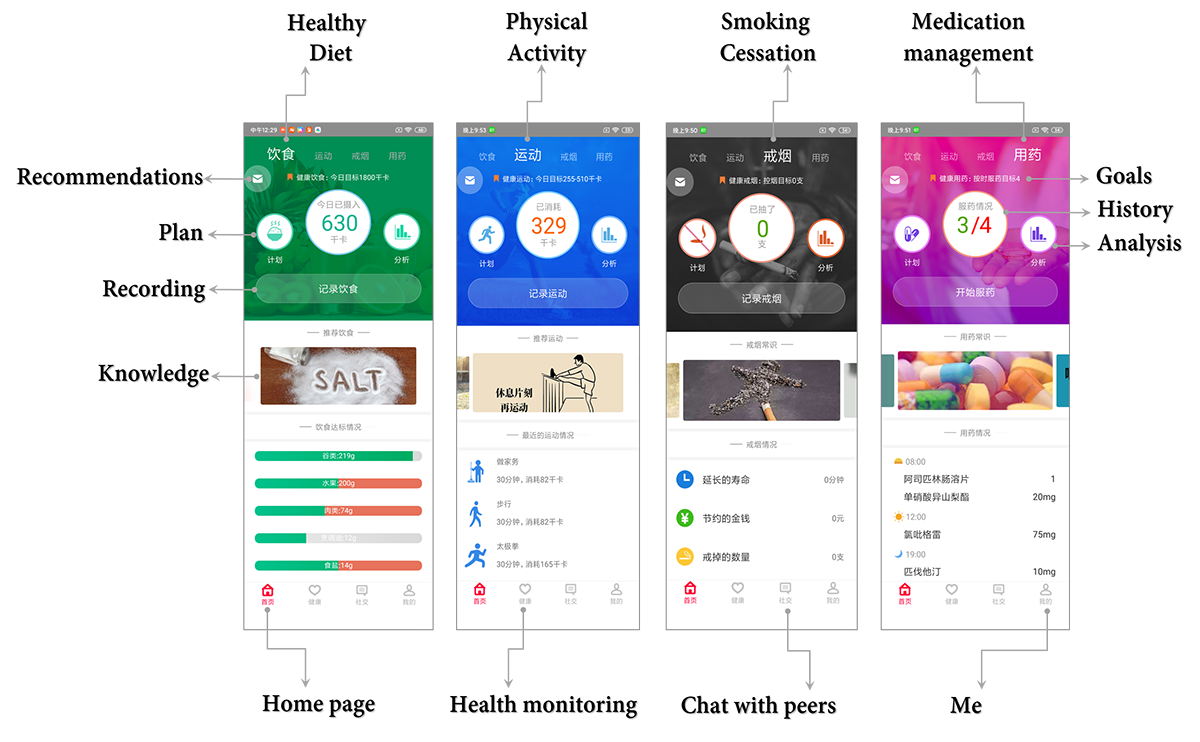
The user interface of the home page of the patient app.

Following the user-centered design principle, the user interfaces for all functional modules were generated by health care professionals. The user interfaces of the patient app’s home page are displayed in Figure 3, which included diet, physical activity, smoking, and medication management with different colors for easy identification. This arrangement allows easy access of the selected modules without complicated manipulation of the app.

**Figure 4:**
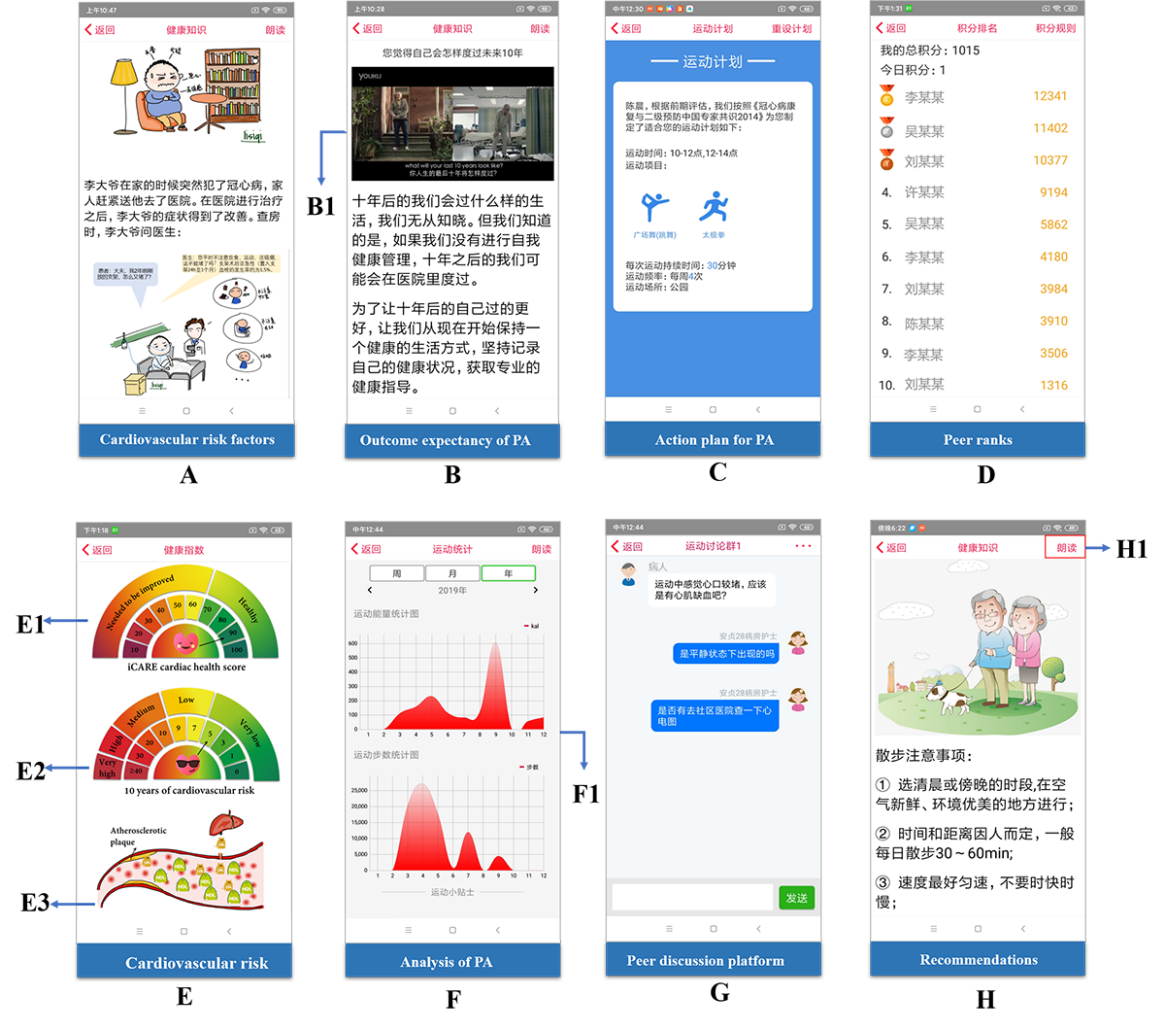
Examples of user interfaces to reflect major moderators and mediators identified within the CAM (Contemplation-Action-Maintenance) model. (A) Risk Perception; (B) Outcome Expectation; (C) Action Planning; (D) Self-efficacy; (E) Perceived Effectiveness; (F) Behavioral Enjoyment; (G) Social Support; (H) Coping Planning. (B1) Video format; (E1) Visualization of iCARE Cardiac Health Score; (E2) Visualization of 10 years of cardiovascular risk; (E3) Visualization of atherosclerotic plaque; (F1) Trend chart; (H1) Read-out mode. iCARE: Intelligent Individualized Cardiovascular App for Risk Elimination; PA: physical activity.

To address the major moderators and mediators identified within the CAM model, techniques were applied to augment the functions of the related features. For example, to increase patient’s risk perception, we applied visual comics to increase individuals’ awareness on potential cardiac events and the severity of a risk (Figure 4A). Visualization was also used to increase patients’ perception on the effectiveness of adhering to healthy behaviors and medication treatment. As shown in Figure 4E, if good adherence was identified on health behaviors, medication adherence, blood pressure, blood glucose, and lipid levels, the Cardiac Health Score will increase. To promote positive outcome expectation, we also used videos, such as visualization of their future life with or without modifying their unhealthy lifestyles, to remind users about the positive effect of following suggested interventions (Figure 4B). Individualized action plans such as plans for physical activity were generated based on patients’ assessment and their preferences, and they were available on the home page for easy reference (Figure 4C). In addition, as shown in Figure 4D, to increase patients’ self-efficacy, a peer rank was designed in iCARE to increase patients’ confidence in maintaining healthy behaviors. We also sent health promotion messages regarding the positive expectations from changing unhealthy behaviors to the patient app. When the patient was at the action stage of behavior change, a predesigned questionnaire was sent to the patient app and patients were asked to respond to the questionnaire to understand their possible barriers. According to their response, iCARE would automatically match the predesigned countermeasures with identified barriers, and push a coping plan to the patient. Patients can revise the coping plan according to their personal features and preferences and form a final coping plan.

### Phase 3: Expert Inspections and Walkthroughs of the iCARE Prototypes

The software engineers translated the user interfaces into prototypes. The finalized alpha version of the iCARE prototype was developed, tested, and discussed among technology professionals to address technical issues throughout the development process. The steps involved in testing the iCARE alpha version are presented in Figure S2 in Multimedia Appendix 1. To verify the basic functionality of the developed modules, engineers tested each module independently with simulated data 10-20 times. To verify the stability of each module after they were finalized, a dedicated software evaluator tested the module 5-10 times with simulated patients’ data.

The beta version of the iCARE prototype was released after the technical issues or bugs identified in the alpha version were resolved. The beta version of iCARE was released in March 10, 2019. An example regarding the logic of the interfaces on medication management is displayed in Figure S3 in Multimedia Appendix 1.

### Phase 4: Usability Testing of iCARE With End Users

#### Overview of Stages

**Figure 5:**
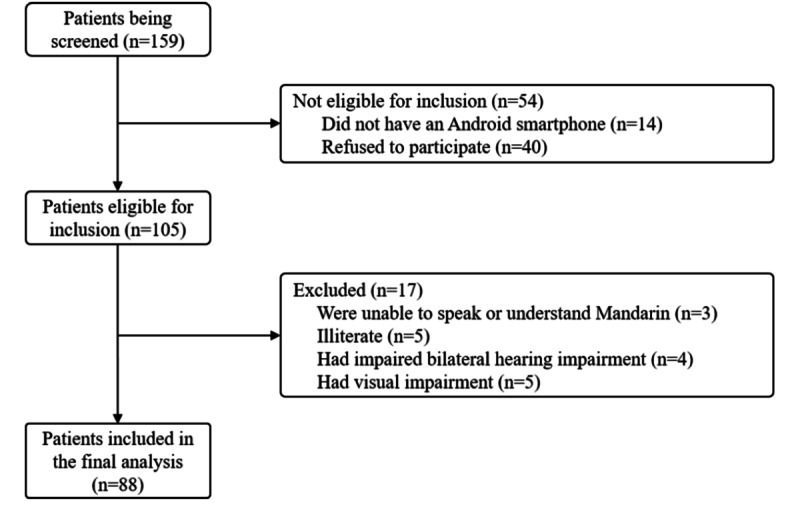
The flow of patient recruitment for the first step usability evaluation study.

After the fully functional iCARE prototype was formulated, we carried out a 2-stage usability testing of the iCARE patient app. Stage I was conducted among 88 patients with CHD after iCARE was released. Figure 5 shows the flowchart of participant recruitment for the first-stage usability testing. A total of 159 patients were diagnosed with CHD during the study period, among which 88 eligible patients participated in the study and were included in the final analysis. There were 40 patients eligible for the study but refused to participate due to various reasons, such as lack of time, conflict of schedule, or simply being uninterested. The basic characteristics of enrolled participants for the first-step usability testing are presented in Table S5 in Multimedia Appendix 1, and they were largely male (72%, 63/88), with a mean age of 60 (SD 9.9) years.

#### Task Analysis

**Figure 6:**
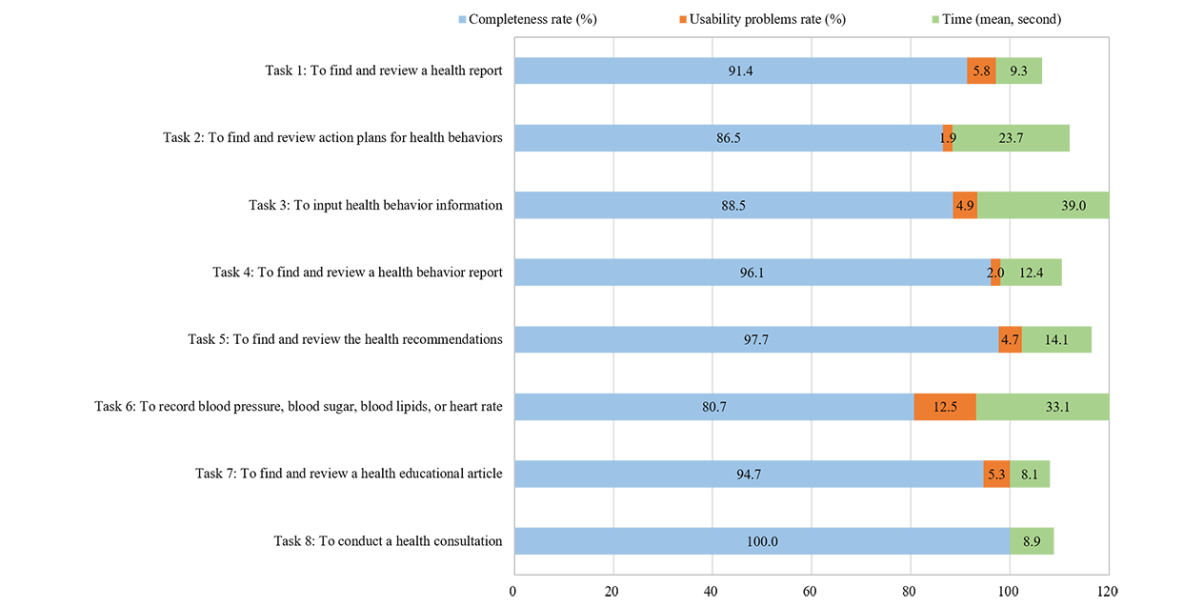
The effectiveness and efficacy evaluation of the patient App based on task analysis.

Figure 6 shows the effectiveness and efficacy of the patient app. Regarding the effectiveness of the app, the results showed that a majority of participants (80%, 70/88) perceived the tasks as easy to complete. The usability problems in completing the 8 tasks were rated from 0% (0/88) to 12.5% (11/88). In terms of the efficacy of the patient app, the average time used to complete tasks 1 through 8 ranged from 8 to 39 seconds.

Table S6 in Multimedia Appendix 1 summarizes the usability problems that emerged from task analysis. A majority (6/17) of the usability problems were related to “ease of input, screen readability, and glanceability” (category 5), which most frequently occurred while entering data for blood pressure, blood sugar, blood lipids, or heart rate (task 6). Regarding the severity of the identified usability problems, 4 were identified as usability catastrophes. For example, regarding task 6 “to record blood pressure, blood sugar, blood lipids, or heart rate,” usability catastrophe was identified when patients reported that the icon was not easy to manipulate in recording blood pressure, as the distance between the 2 circles was too close and it was hard to manipulate.

#### Usability Evaluation With the Health-ITUES

Table 1 illustrates participants’ perceptions on using iCARE in the first-stage usability testing. The mean total score of the Health-ITUES among participants was 90.1 (IQR 83.3-99.0), with a mean score on each item at 4.5 (SD 0.50). The mean scores of items related to the impact, perceived usefulness, perceived ease of use, and user control of the patient app ranged from 4.3 to 4.6. Meanwhile, based on patients’ response to items 7, 9, and 14 of the Health-ITUES, 90% (79/88), 94% (83/88), and 82% (72/88) of enrolled patients perceived iCARE as satisfactory, useful, and easy to use, respectively.

After completion of stage I usability testing, the iCARE beta version was updated and revised based on the results of initial usability evaluation and task analysis. Such revisions included, but not limited to, increase of font sizes, modification of icons (ie, increasing the distance between the 2 circles of the icon for blood pressure), and utilization of more colors for easy identification in related user interfaces. The finalized iCARE version was released and used in the RCT study. Stage II usability testing was conducted among 61 (85% [52/61] male, mean age 53 [SD 8.2] years) individuals in the RCT intervention arm after they used the app for 6 months. The mean total score of the Health-ITUES was 89.0 (IQR 77.0-99.5), with the mean score on each item being 4.3 (SD 0.64). The mean scores of items related to the subdimensions (impact, perceived usefulness, perceived ease of use, and user control) ranged from 4.2 to 4.5. Among enrolled participants, 89% (54/61) were satisfied with the use of the app, 93% (57/61) perceived the app as useful, and 70% (43/61) as easy to use, based on patients’ response to related items (7, 9, and 14). In addition, 52% (32/61) and 11% (7/61) reported using the patient app at least once a week or every day, respectively.

As shown in Figure S4 in Multimedia Appendix 1, no significant changes were found in terms of the total score of the Health-ITUES (*P*=.25) as well as the 4 dimensions of the Health-ITUES (*P*=.52, .68, .14, and .10 for impact, perceived usefulness, perceived ease of use, and user control, respectively) between participants in the first and second stages of usability testing. There was also no significant difference identified in terms of the rates of satisfaction (*P*=.85), usefulness (*P*=.86), and ease of use (*P*=.12) between first and second usability evaluations.

The number of times individuals accessed the patient app showed that they used it for a mean of 33.5 (SD 75.3) times over 6 months. The most frequently visited screens were related to health recommendation, followed by daily monitoring of health indicators, health behaviors, and medication adherence (26179, 1649, and 942 times, respectively). There were no significant differences in the usage of the patient app among patients in terms of their working status (retired verses not retired; *P*=.13), educational levels (*P*=.34), and gender (*P*=.97). In stage II usability study, 38/61 patients used the smart watch, and 23 patients did not use the smart watch. The mean usage of the app among patients who used the smart watch was higher than those who did not (97.7 vs. 12.5, *P*<.001) over 6 months. As the blood glucose monitor was not distributed to patients with diabetes (10/61 patients) in stage II testing, it was not available for testing the differences in this regard.

**Table 1:** Summary of the Health-ITUES^a^ questionnaire.

Items		First testing, mean (SD)	Second testing, mean (SD)
**Impact (Cronbach α: .863)**		4.6 (0.62)	4.5 (0.76)
	1. I think iCARE^b^ can be a positive addition for heart health for patients living with CHD^c^.	4.6 (0.71)	4.4 (0.74)
	2. I think iCARE can improve the quality of life of persons living with CHD.	4.6 (0.61)	4.6 (0.61)
	3. iCARE is an important part of meeting my information needs related to self-management of CHD-related risk factors.	4.5 (0.76)	4.4 (0.96)
**Perceived usefulness (Cronbach α: .901)**		4.6 (0.51)	4.4 (0.63)
	4. Using iCARE makes it easier to self-manage my CHD-related risk factors.	4.5 (0.70)	4.5 (0.67)
	5. Using iCARE enables me to self-manage my CHD-related risk factors more quickly.	4.6 (0.60)	4.5 (0.67)
	6. Using iCARE makes it more likely that I can self-manage my iCARE- related risk factors.	4.6 (0.60)	4.4 (0.88)
	7. Using iCARE is useful for self-management of CHD-related risk factors.	4.6 (0.70)	4.6 (0.62)
	8. I think iCARE presents a more equitable process for self-management of CHD-related risk factors.	4.6 (0.70)	4.5 (0.72)
	9. I am satisfied with iCARE for self-management of CHD-related risk factors.	4.6 (0.68)	4.5 (0.74)
	10. I self-manage my CHD-related risk factors in a timely manner because of iCARE.	4.5 (0.77)	4.3 (0.83)
	11. Using iCARE increases my ability to self-manage my CHD-related risk factors.	4.6 (0.63)	4.4 (0.74)
	12. I am able to self-manage my CHD-related risk factors whenever I use iCARE.	4.4 (0.78)	4.3 (0.83)
**Perceived ease of use (Cronbach α: .899)**		4.3 (0.76)	4.2 (0.76)
	13. I am comfortable with my ability to use iCARE.	4.3 (0.88)	4.3 (0.73)
	14. Learning to operate iCARE is easy for me.	4.3 (0.90)	4.0 (1.05)
	15. It is easy for me to become skillful at using iCARE.	4.3 (0.95)	4.0 (1.06)
	16. I find iCARE easy to use.	4.5 (0.77)	4.2 (0.96)
	17. I can always remember how to log on to and use iCARE.	4.3 (0.97)	4.3 (0.85)
**User control (Cronbach α: .743)**		4.4 (0.67)	4.2 (0.81)
	18. iCARE gives error messages that clearly tell me how to fix problems.	4.3 (0.91)	4.2 (0.90)
	19. Whenever I make a mistake using iCARE, I recover easily and quickly.	4.4 (0.89)	4.2 (0.88)
	20. The information (such as online help, on-screen messages, and other documentation) provided with iCARE is clear.	4.7 (0.60)	4.2 (0.96)

^a^The Modified Health-Information Technology Usability Survey was adopted from Schnall et al [43]. ©[2021] Capital Medical University, Beijing, China. All rights reserved. Adapted with permission from the “Health IT Usability Evaluation Scale.”

^b^iCARE: Individualized, Intelligent and Integrated Cardiovascular App for Risk Elimination.

^c^CHD: coronary heart disease.

## Discussion

### Principal Findings

In this study, we developed an intelligent and individualized health care management system for individuals with CHD, iCARE, following a 4-phase user-centered approach. iCARE was designed to facilitate patients’ adherence to multiple health behaviors (diet, physical activity, and smoking) and preventive medications. The design and development of iCARE were realized through incorporation of individualized interventions, which were developed through a theory-driven and evidence-based approach and following the principles of the nursing process. iCARE was developed to reflect designated interventional strategies that target the mediators and moderators of the CAM model by using an individualized approach and visualization techniques. iCARE is an intelligent health management system that has a set of “IF–THEN” algorithms that trigger real-time monitoring, person-centered care, and automatic tailored feedback, based on data from initial assessment and health monitoring via manual entry or wearable devices. iCARE includes functions in facilitating health assessment and monitoring, health behavior and medication management, intervention implementation, and health counseling. The results of the 2-stage usability testing revealed that iCARE has acceptable usability. The patient app was perceived as highly acceptable among individuals with CHD, with a majority perceiving that the app was satisfactory, useful, and easy to use.

### Comparison With Prior Work

Evidence has shown that lack of scalability in health care management system can hamper system growth and prevent it from providing person-centered care [48]. A health care system adopting multiple services, such as iCARE, requires software developers to build and deploy the system in a reliable and timely fashion. Such system should be scalable to adapt to the evolving needs of patients and health care professionals [48]. The key barrier to achieve this is the selection of a suitable software architecture [36]. In this study, the MicroService architecture [49], an emerging architectural style for developing distributed apps, was applied in the development of iCARE. With the application of this architecture, our system may have the advantages in realizing availability, flexibility, scalability, and allowing multiple services to be scaled up independently [49,50]. Implementation of the MicroService architecture in this study allowed us to develop, modify, and deploy iCARE promptly based on the needs identified in the task analysis and issues revealed during the first and second usability evaluations.

Overall, the iCARE patient app was perceived as useful and satisfactory despite several commonly identified usability problems that were determined to be similar to those of other health-related self-management apps [51]. The 2-stage usability evaluation revealed that the mean scores on each item of the Health-ITUES were all above 4.0, and the end users commented that iCARE had much better usability in terms of perceived usefulness. There are multiple reasons for this: First, the development of the iCARE interventions was evidence based and followed a theory-driven approach. Second, iCARE was designed based on the user-centered design principle, which was shown to be effective in increasing user’s engagement, thereby making the interactive system useful for targeted end users [35]. In our preliminary work, we carefully assessed the needs and preferences of individuals with CHD not only during the user interface design phase, but also during the development and modification phases. In addition, as suggested by Dawson et al [28], to promote sustained and significant behavior change among users, we encouraged nurses and other health professionals to be actively involved in the inspections and walkthroughs of the iCARE prototypes. Second, as outlined in our previous work [33], to ensure the interventions of iCARE are evidence based and compliant with clinical guidelines, we structured them based on available cardiovascular secondary prevention guidelines [3,30,31]. Meanwhile, an integrated behavior change theory, the CAM model, was used to guide the design of iCARE, to ensure interventions are empirically based [33]. Third, based on a previous study [52], a systematic and problem-solving approach along with a patient-centered and goal-oriented method was used in designing our system. To ensure the functions of iCARE are acceptable and useful, the components and schematic diagram of iCARE were designed following the principles of the nursing process, which are identified as essential to ensure nurses deliver holistic and patient-centered care [35]. These may have contributed to the good functionality of iCARE in this study.

Meanwhile, the perceived ease of use of a system by users is another essential element in developing useful tools [53]. However, achieving the balance between usefulness and ease of use is challenging as these 2 components are often contradictory during app development [54]. When designing a system, some designers sacrifice usefulness to provide ease of use, or vice versa [54]. For the health management of individuals with CHD, comprehensive CHD prevention strategies were recommended by the cardiovascular secondary prevention guidelines [3,30,31]. Therefore, the system should be designed with multiple functionalities to incorporate comprehensive CHD prevention strategies to achieve its optimal usefulness. However, increasing the amount of functionality in a system may also increase the treatment-related burden on patients and affect effective care delivery [55]. This is reflected in our case as well: the multiple functionalities included in our system increased the complexity of the system, which might have contributed to the lower scores on the ease of use functionality of the patient app during the first and second usability testing phases.

Strategies to address the ease-of-use functionality of the system are vital to reduce unnecessary burden on individuals. As identified by Tsopra et al [56], interfaces designed following usability principles suggested by the Healthcare Information and Management Systems Society [57], including effective information presentation, consistency, efficient interactions, effective use of language, and minimizing cognitive load, were perceived as ease of use. In our system, we applied several techniques to improve its ease of use, including the following: (1) the use of larger font sizes and different colors to improve readability; (2) the use of a read-out mode to minimize individuals’ cognitive load; and (3) limiting the amount of text message and replacing it with figures, infographics, and chart to ensure effective and visual information presentation. These techniques are also in agreement with a previous finding [58], in which the use of color-coded visual layout improved end user’s perceived ease of use for a system. The findings of the usability testing in our study indicated that most participants perceived the patient app as easy to use.

However, we are fully aware that some design and functional elements of the app still need to be addressed to improve its ease of use. For example, the number of interfaces on the patient app should be reduced to promote efficient interactions; some icons should be designed as simpler and meaningful as possible to be easily recognized; and the system should provide more guidance and assistance to end users to avoid human errors and increase their perceived user control. In addition to the lessons learned during our system designing and developing process, future studies should pay more attention to maximize the learnability component through minimizing cognitive loads during the designing process, and improve the memorability of the system that can assist users to reestablish proficiency after a long period of inactivity [59].

### Limitations

This study has several limitations. First, given the nature of the study, participants who were willing to participate in this study may be more motivated and more proficient in using the mHealth apps than the general population. Second, this study was conducted among patients who were admitted to hospitals in Beijing, which is a technology-advanced city in China. However, the 2 study hospitals are the most recognized hospitals in cardiovascular disease care in China, and patients admitted to these hospitals are from all over the country, including those from rural and underserved areas. Third, in the second stage of usability testing, we only subjectively evaluated the usability of the patient app; therefore, some usability problems within our system may not have been discovered adequately. Fourth, although the needs assessment was analyzed among patients with CHD, they were not actively involved in the interface designing process, which might influence the usability of the patient app. However, their response to stage I usability testing was applied to modify the system, and the updated version was used in the RCT and second usability evaluation. Fifth, the care provider app only has a few basic functions in its current stage; therefore, its usability was not considered in this study. However, we will expand the care provider app to include more fully functional components and evaluate its usability in the future. In addition, in its current stage, the data from external devices cannot be automatically uploaded to the patient app which might influence its usability. Finally, the iCARE system was developed to be only compatible with Android-based smartphones, and thus patients who used an iOS-based smartphones were excluded. Although iPhones are popular in China, they are very expensive, and are considered as high-end mobile phones that are similar to the high-end Huawei mobile phones; besides, iPhone users are usually mid- or high-income population that live in tech-developed cities, such as Shanghai or Beijing. Moreover, after completing our RCT, we will develop an updated iCARE system that will be compatible with both Android and iOS versions, as many patients who used iPhones expressed their interest and intention in using iCARE to manage their diseases and health. Therefore, we will perform additional usability testing in a more representative population.

### Conclusions

This study developed the iCARE system with the aim to facilitate patients’ adherence to multiple health behaviors and preventive medications through incorporation of theory-driven, evidence-based, individualized interventions and tailored automatic feedback, following the CAM model and principles of the nursing process. iCARE was demonstrated to be highly satisfactory, useful, and easy to use among individuals with CHD and had acceptable usability. We are currently evaluating the effectiveness of iCARE in patients with CHD via a randomized clinical trial to better understand the real-time usability of the app, as well as patients’ experience in using the app. In future studies, we will revise iCARE based on the findings of this study and develop a version that is compatible with iOS-based smartphones.

## Appendix

Multimedia Appendix 1 Preliminary work, study participants and procedures, supplementary Tables and Figures.

Multimedia Appendix 2 Task 1.

Multimedia Appendix 3 Task 2.

Multimedia Appendix 4 Task 3.

Multimedia Appendix 5 Task 4.

Multimedia Appendix 6 Task 5.

Multimedia Appendix 7 Task 6.

Multimedia Appendix 8 Task 7.

Multimedia Appendix 9 Task 8.

## Abbreviations

CAM: Contemplation–Action–Maintenance model
CHD: coronary heart disease
Health-ITUES: Health Information Technology Usability Survey
iCARE: Intelligent Individualized Cardiovascular App for Risk Elimination
ISO: International Organization for Standardization
mHealth: mobile health
RCT: randomized controlled trial

## References

[1] Mendis S, Puska PB, Norrving B (2011). Global Atlas on Cardiovascular Disease Prevention and Control.

[2] Gaziano TA, Bitton A, Anand S, Abrahams-Gessel S, Murphy A (2010). Growing epidemic of coronary heart disease in low- and middle-income countries. Curr Probl Cardiol.

[3] National Center for Cardiovascular Disease of China (2020). Report on Cardiovascular Disease in China 2019 (in Chinese).

[4] Devaraj Sm, Kriska Am, Orchard Tj, Miller Rg, Costacou T (2021). Cardiovascular health in early adulthood predicts the development of coronary heart disease in individuals with type 1 diabetes: 25 year follow-up from the Pittsburgh Epidemiology of Diabetes Complications study. Diabetologia.

[5] Lv J, Yu C, Guo Y, Bian Z, Yang L, Chen Y, Tang X, Zhang W, Qian Y, Huang Y, Wang X, Chen J, Chen Z, Qi L, Li L, China Kadoorie Biobank Collaborative Group (2017). Adherence to healthy lifestyle and cardiovascular diseases in the Chinese population. J Am Coll Cardiol.

[6] Ford ES, Ajani UA, Croft JB, Critchley JA, Labarthe DR, Kottke TE, Giles WH, Capewell S (2007). Explaining the decrease in U.S. deaths from coronary disease, 1980-2000. N Engl J Med.

[7] Han C, Liu F, Yang X, Chen J, Li J, Cao J, Li Y, Shen C, Yu L, Liu Z, Wu X, Zhao L, Hu D, Lu X, Wu X, Gu D (2018). Ideal cardiovascular health and incidence of atherosclerotic cardiovascular disease among Chinese adults: the China-PAR project. Sci China Life Sci.

[8] Hu G, Zhou M, Liu J, Smith SC, Ma C, Ge J, Huo Y, Fonarow GC, Hao Y, Liu J, Taubert KA, Morgan L, Yang N, Zeng Y, Han Y, Zhao D, CCC-ACS Investigators (2020). Smoking and provision of smoking cessation interventions among inpatients with acute coronary syndrome in China: Findings from the Improving Care for Cardiovascular Disease in China-Acute Coronary Syndrome Project. Glob Heart.

[9] Du H, Li L, Bennett D, Guo Y, Key TJ, Bian Z, Sherliker P, Gao H, Chen Y, Yang L, Chen J, Wang S, Du R, Su H, Collins R, Peto R, Chen Z, China Kadoorie Biobank Study (2016). Fresh fruit consumption and major cardiovascular disease in China. N Engl J Med.

[10] Kotseva K, Wood D, De Bacquer D, De Backer G, Rydén L, Jennings C, Gyberg V, Amouyel P, Bruthans J, Castro Conde A, Cífková R, Deckers JW, De Sutter J, Dilic M, Dolzhenko M, Erglis A, Fras Z, Gaita D, Gotcheva N, Goudevenos J, Heuschmann P, Laucevicius A, Lehto S, Lovic D, Miličić D, Moore D, Nicolaides E, Oganov R, Pajak A, Pogosova N, Reiner Z, Stagmo M, Störk S, Tokgözoğlu L, Vulic D, EUROASPIRE Investigators (2016). EUROASPIRE IV: A European Society of Cardiology survey on the lifestyle, risk factor and therapeutic management of coronary patients from 24 European countries. Eur J Prev Cardiol.

[11] Tang L, Patao C, Chuang J, Wong ND (2013). Cardiovascular risk factor control and adherence to recommended lifestyle and medical therapies in persons with coronary heart disease (from the National Health and Nutrition Examination Survey 2007-2010). Am J Cardiol.

[12] Teo K, Lear S, Islam S, Mony P, Dehghan M, Li W, Rosengren A, Lopez-Jaramillo P, Diaz R, Oliveira G, Miskan M, Rangarajan S, Iqbal R, Ilow R, Puone T, Bahonar A, Gulec S, Darwish EA, Lanas F, Vijaykumar K, Rahman O, Chifamba J, Hou Y, Li N, Yusuf S, PURE Investigators (2013). Prevalence of a healthy lifestyle among individuals with cardiovascular disease in high-, middle- and low-income countries: The Prospective Urban Rural Epidemiology (PURE) study. JAMA.

[13] Wood DA, Kotseva K, Connolly S, Jennings C, Mead A, Jones J, Holden A, De Bacquer D, Collier T, De Backer G, Faergeman O, EUROACTION Study Group (2008). Nurse-coordinated multidisciplinary, family-based cardiovascular disease prevention programme (EUROACTION) for patients with coronary heart disease and asymptomatic individuals at high risk of cardiovascular disease: a paired, cluster-randomised controlled trial. Lancet.

[14] Rautio N, Jokelainen J, Pölönen A, Oksa H, Peltonen M, Vanhala M, Puolijoki H, Moilanen L, Tuomilehto J, Uusitupa M, Keinänen-Kiukaanniemi S, Saaristo T (2015). Changes in lifestyle modestly reduce the estimated cardiovascular disease risk in one-year follow-up of the Finnish diabetes prevention program (FIN-D2D). Eur J Cardiovasc Nurs.

[15] Lv J, Liu Q, Ren Y, He P, Wang S, Gao F, Li L, Community Interventions for Health (CIH) collaboration (2014). A community-based multilevel intervention for smoking, physical activity and diet: short-term findings from the Community Interventions for Health programme in Hangzhou, China. J Epidemiol Community Health.

[16] Du H, Venkatakrishnan A, Youngblood GM, Ram A, Pirolli P (2016). A group-based mobile application to increase adherence in exercise and nutrition programs: a factorial design feasibility study. JMIR Mhealth Uhealth.

[17] Maron DJ, Mancini GBJ, Hartigan PM, Spertus JA, Sedlis SP, Kostuk WJ, Berman DS, Teo KK, Weintraub WS, Boden WE, COURAGE Trial Group (2018). Healthy behavior, risk factor control, and survival in the COURAGE trial. J Am Coll Cardiol.

[18] Milne-Ives M, Lam C, De Cock C, Van Velthoven MH, Meinert E (2020). Mobile apps for health behavior change in physical activity, diet, drug and alcohol use, and mental health: systematic review. JMIR Mhealth Uhealth.

[19] Hong YA, Zhou Z, Fang Y (2017). Digital divide and health disparities in China: data from a national longitudinal survey of CHARLS. Stud Health Technol Inform.

[20] Sobrinho A, da Silva LD, Perkusich A, Pinheiro ME, Cunha P (2018). Design and evaluation of a mobile application to assist the self-monitoring of the chronic kidney disease in developing countries. BMC Med Inform Decis Mak.

[21] Bondaronek P, Alkhaldi G, Slee A, Hamilton FL, Murray E (2018). Quality of publicly available physical activity apps: review and content analysis. JMIR Mhealth Uhealth.

[22] Ferrara G, Kim J, Lin S, Hua J, Seto E (2019). A focused review of smartphone diet-tracking apps: usability, functionality, coherence with behavior change theory, and comparative validity of nutrient intake and energy estimates. JMIR Mhealth Uhealth.

[23] Hoeppner BB, Hoeppner SS, Seaboyer L, Schick MR, Wu GWY, Bergman BG, Kelly JF (2016). How smart are smartphone apps for smoking cessation? A content analysis. Nicotine Tob Res.

[24] McKay FH, Wright A, Shill J, Stephens H, Uccellini M (2019). Using health and well-being apps for behavior change: a systematic search and rating of apps. JMIR Mhealth Uhealth.

[25] Morrissey EC, Corbett TK, Walsh JC, Molloy GJ (2016). Behavior change techniques in apps for medication adherence: a content analysis. Am J Prev Med.

[26] Kumar N, Khunger M, Gupta A, Garg N (2015). A content analysis of smartphone-based applications for hypertension management. J Am Soc Hypertens.

[27] Xiao Q, Lu S, Wang Y, Sun L, Wu Y (2017). Current status of cardiovascular disease-related smartphone apps downloadable in China. Telemed J E Health.

[28] Xie Bo, Su Zhaohui, Zhang Wenhui, Cai Run (2017). Chinese cardiovascular disease mobile apps' information types, information quality, and interactive functions for self-management: systematic review. JMIR Mhealth Uhealth.

[29] Han Myeunghee, Lee Eunjoo (2018). Effectiveness of mobile health application use to improve health behavior changes: a systematicrReview of randomized controlled trials. Healthc Inform Res.

[30] Piepoli MF, Hoes AW, Agewall S, Albus C, Brotons C, Catapano AL, Cooney M, Corrà U, Cosyns B, Deaton C, Graham I, Hall MS, Hobbs FDR, Løchen M, Löllgen H, Marques-Vidal P, Perk J, Prescott E, Redon J, Richter DJ, Sattar N, Smulders Y, Tiberi M, van DWHB, van DI, Verschuren WMM, Binno S, ESC Scientific Document Group (2016). 2016 European Guidelines on cardiovascular disease prevention in clinical practice: The Sixth Joint Task Force of the European Society of Cardiology and Other Societies on Cardiovascular Disease Prevention in Clinical Practice (constituted by representatives of 10 societies and by invited experts) Developed with the special contribution of the European Association for Cardiovascular Prevention & Rehabilitation (EACPR). Eur Heart J.

[31] Smith SC, Benjamin EJ, Bonow RO, Braun LT, Creager MA, Franklin BA, Gibbons RJ, Grundy SM, Hiratzka LF, Jones DW, Lloyd-Jones DM, Minissian M, Mosca L, Peterson ED, Sacco RL, Spertus J, Stein JH, Taubert KA (2011). AHA/ACCF secondary prevention and risk reduction therapy for patients with coronary and other atherosclerotic vascular disease: 2011 update: a guideline from the American Heart Association and American College of Cardiology Foundation endorsed by the World Heart Federation and the Preventive Cardiovascular Nurses Association. J Am Coll Cardiol.

[32] Bartholomew LC, Markham CM, Ruiter RAC, Fernandez ME, Kok G, Parcel GS (2016). Planning Health Promotion Programs: An Intervention Mapping Approach.

[33] Chen Y, Wu F, Wu Y, Li J, Yue P, Deng Y, Lamb KV, Fong S, Liu Y, Zhang Y (2019). Development of interventions for an intelligent and individualized mobile health care system to promote healthy diet and physical activity: using an intervention mapping framework. BMC Public Health.

[34] Yue P, Wu Y, Zhang Y, Chen Y, Li J, Xu Y, Liu Y (2021). Contemplation-action-maintenance model of behaviour change for persons with coronary heart disease: A qualitative study. J Clin Nurs.

[35] Dawson RM, Felder TM, Donevant SB, McDonnell KK, Card EB, King CC, Heiney SP (2020). What makes a good health 'app'? Identifying the strengths and limitations of existing mobile application evaluation tools. Nurs Inq.

[36] Chen L (2018). Microservices: Architecting for continuous delivery and DevOps.

[37] Yang Y, Zu Q, Liu P, Ouyang D, Li X (2018). MicroShare: Privacy-preserved medical resource sharing through MicroService architecture. Int J Biol Sci.

[38] Taherizadeh S, Stankovski V, Grobelnik M (2018). A capillary computing architecture for dynamic internet of things: orchestration of microservices from Edge devices to Fog and Cloud providers. Sensors (Basel).

[39] The App Design App.

[40] Lumsden J (2008). Handbook of Research on User Interface Design and Evaluation for Mobile Technology.

[41] MOBGEN High-Fidelity Prototyping: What, When, Why and How?.

[42] Kushniruk AW, Borycki EM (2015). Integrating low-cost rapid usability testing into agile system development of healthcare IT: a methodological perspective. Stud Health Technol Inform.

[43] Schnall R, Cho H, Liu J (2018). Health Information Technology Usability Evaluation Scale (Health-ITUES) for usability assessment of mobile health technology: validation study. JMIR Mhealth Uhealth.

[44] (1998). Ergonomic Requirements for Office Work With Visual Display Terminals (VDTs)-Part 11: Guidance on Usability.

[45] Maramba I, Chatterjee A, Newman C (2019). Methods of usability testing in the development of eHealth applications: A scoping review. Int J Med Inform.

[46] Nielsen J (1994). Severity Ratings for Usability Problems.

[47] Vélez O, Okyere PB, Kanter AS, Bakken S (2014). A usability study of a mobile health application for rural Ghanaian midwives. J Midwifery Womens Health.

[48] Roca S, Sancho J, García J, Alesanco (2020). Microservice chatbot architecture for chronic patient support. J Biomed Inform.

[49] Newman S (2021). Building Microservices: Designing Fine-Grained Systems.

[50] Garcia-Moreno FM, Bermudez-Edo M, Garrido JL, Rodríguez-García Estefanía, Pérez-Mármol José Manuel, Rodríguez-Fórtiz María José (2020). A microservices e-health system for ecological frailty assessment using wearables. Sensors (Basel).

[51] Fu H, McMahon SK, Gross CR, Adam TJ, Wyman JF (2017). Usability and clinical efficacy of diabetes mobile applications for adults with type 2 diabetes: A systematic review. Diabetes Res Clin Pract.

[52] Kim H, Park H, Min YH, Jeon E (2013). Development of an obesity management ontology based on the nursing process for the mobile-device domain. J Med Internet Res.

[53] Davis FD, Bagozzi RP, Warshaw PR (1989). User acceptance of computer technology: a comparison of two theoretical models. Management Science.

[54] Ease of Use.

[55] Tran V, Barnes C, Montori VM, Falissard B, Ravaud P (2015). Taxonomy of the burden of treatment: a multi-country web-based qualitative study of patients with chronic conditions. BMC Med.

[56] Tsopra R, Jais J, Venot A, Duclos C (2014). Comparison of two kinds of interface, based on guided navigation or usability principles, for improving the adoption of computerized decision support systems: application to the prescription of antibiotics. J Am Med Inform Assoc.

[57] Belden J, Grayson R, Barnes J (2009). Defining and Testing EMR Usability: Principles and Proposed Methods of EMR Usability Evaluation and Rating.

[58] Lazard AJ (2020). Design cues for tobacco communication: Heuristic interpretations and usability of online health information about harmful chemicals. Int J Med Inform.

[59] Shukairy A 5 usability design tips for a better user experience design.

