# Supplementary material for: An Intelligent Individualized Cardiovascular App for Risk Elimination (iCARE) for Individuals With Coronary Heart Disease: Development and Usability Testing Analysis
Source: JMIR Mhealth Uhealth. 2021 Dec 13;9(12):e26439. doi: 10.2196/26439 (PMC8713096; doi:10.2196/26439)
Supplement: Multimedia Appendix 1 [file mhealth_v9i12e26439_app1.docx]

**Multimedia Appendix 1**

**Preliminary Work**

**Patients’ needs assessment**

To design an effective iCARE system that meets patients’ needs, we conducted a need assessment for the use of mHealth-based system which was described elsewhere [1]. In brief, the needs assessment was conducted through literature review, in-depth interviews (six patients with CHD) and focus group discussions (two focus groups). The results of the needs assessment [1] suggested that the main themes of patients’ needs were developing mutually agreed action plans for management of health behaviors and medications, continuously monitoring health status, providing health status alert as well as tailored interventions and health knowledge. They also suggested that the contents of the intervention should use visual formats with easily recognizable configurations including contrasting colors, distinctive graphical shapes etc. along with the use of voice, videos.

**Intervention formulation and algorithm development**

To increase the effectiveness of the iCARE system on changing unhealthy behaviors, the contents of the intervention was formulated based on existing evidence-based health behavior change strategies from high-quality studies and perspectives of healthcare professionals as described in our earlier work [1]. In summary, a set of iCARE interventions that were designed to improve patients’ adherence to health behaviors and preventive medications were developed based on the Intervention Mapping Framework (IMF) [2] and the CAM behavior change model [3]. The IMF comprises a stepwise process to develop, implement, and evaluate behavioral change interventions [3]. The CAM is an integrated behavioral change model that was modified based on the Health Action Process Approach model and focuses on the characteristics of different stages of behavior change among patients with CHD [3]. In addition, the iCARE system incorporated multi-facet interventions in reflecting health behavior changes and medication management. To ensure patients receiving tailored feedbacks and recommendations, the interventions also followed the “IF-THEN” algorithm. The algorithms were developed by a set of rule engine with a logical combination of various rules. The rules include patients’ initial assessment data (i.e. diagnosis, whether the patient undergo PCI, current risk factors, stages of behavior change, mediators and moderators from CAM model) and the dynamic changes of health data acquired from continuous monitoring (i.e. health behaviors, medication adherence, blood pressure).


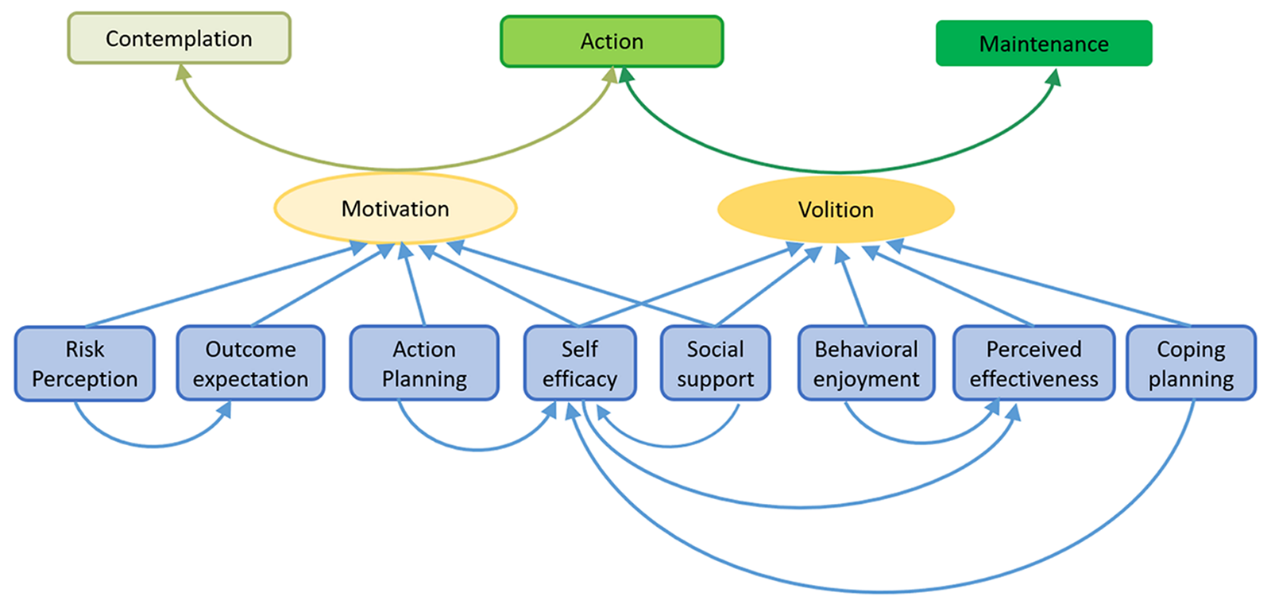


The Contemplation-Action-Maintenance (CAM) model [1, 3]

**References**

1. Chen Y, Wu F, Wu Y, Li J, Yue P, Deng Y, Lamb KV, Fong S, Liu Y, Zhang Y. Development of interventions for an intelligent and individualized mobile health care system to promote healthy diet and physical activity: using an intervention mapping framework. BMC public health 2019, 19:1311. PMID: 31623589. DOI: 10.1186/s12889-019-7639-7.

2. Bartholomew LK, C.M, Markham CM, Ruiter RAC, Fernandez ME, Kok G, Parcel GS. Planning Health Promotion Programs: An Intervention Mapping Approach. John Wiley & Sons 2016.

3. Yue P, Wu Y, Zhang Y, Chen Y, Li J, Xu Y, and Liu Y. Contemplation-action-maintenance model of behaviour change for persons with coronary heart disease: A qualitative study. J Clin Nurs. 2021 May;30(9-10):1464-1478. doi: 10.1111/jocn.15699. Epub 2021 Mar 11. PMID: 33555622.

**Usability Testing: Study Participants and Procedures**

**Study participants**

Following convenient sampling, we recruited eligible participants from three cardiac wards of two university-affiliated hospitals in Beijing, China. The inclusion criteria of the study were: patients who had a documented diagnosis of CHD including history of AMI, ACS, or PCI either as emergency or elective procedures; had at least one smartphone with Android system and used daily; reported with at least one unhealthy behavior among diet, physical activity, smoking, and nonadherence to medication; and were 18 years or older. Exclusion criteria of the study were: patients who had a history of psychiatric and severe neurological disorders that affected patients’ cognitive function; were unable to speak or understand Mandarin, or illiterate; and had impaired bilateral hearing, or visual impairment which limited the use of the smartphone.

**Procedures**

Four nursing investigators in the study team screened patients’ medical records and identified eligible patients according to the inclusion and exclusion criteria. Trainings on using the apps and the procedures for usability testing were provided to the investigators according to the study protocol. The investigators approached eligible patients and explained the aims of the study. Training videos about the patient apps were shown to all patients.

**Examples: Instant and individualized feedback and tailored recommendations for changing behavior based on the built-in algorithms**

Example 1 [1]: IF “Mr. Wang’s physical activity was in the action stage, and the patient has walked < 6500 steps during the day”, THEN the text-message and a link to a questionnaire is sent: “Mr. Wang, your physical activity report shows that you have done well in recent days, but you did not make much progress today. I guess there were some causes for this. Please answer the questionnaire, so we can try to understand the reason that you did not keep up with your regular physical activity”. IF “the patient chooses the cause: I could not go out to do exercise because of the bad weather”, THEN a coping plan is sent: “Please, watch the video. You can find some indoor activities that may be suitable for you.”

Example 2 [1]: IF “the patient was in contemplation stage for diet and she/he had a salt intake >5 g/d, or/and cooking oil intake > 25 g/d, and a low level of outcome expectation”, THEN a message is sent that reads “Mr. Wang, do you want to know the benefits of low salt and low-fat diets? Please go watch the comic.”

Reference:

[1] Chen Y, Wu F, Wu Y, Li J, Yue P, Deng Y, Lamb KV, Fong S, Liu Y and Zhang Y. Development of interventions for an intelligent and individualized mobile health care system to promote healthy diet and physical activity: using an intervention mapping framework. BMC Public Health. 2019;19:1311. PMID: 31623589. DOI: 10.1186/s12889-019-7639-7.

**Table S1. International and national data standards used to develop the iCARE system**

|  | **Standard Name** | **Standards** | **Standards number** |
| --- | --- | --- | --- |
| 1 | International Classification of Diseases Code (ICD-10) | National standard | GB/T14396 |
| 2 | Country name code | National standard | GB26599 |
| 3 | National Code | National standard | GB3304-91 |
| 4 | Device name code | National standard | WZB01-90 |
| 5 | Area code | National standard | DB2260 |
| 6 | Professional technical job code | National standard | GB8561 |
| 7 | Language code | National standard | GB4880 |
| 8 | Occupation code | National standard | GB6565-86 |
| 9 | Administrative job code | National standard | GB12403 |
| 10 | Administrative level code | National standard | GB12407 |
| 11 | Job category code | National standard | GB/T14949-94 |
| 12 | Position classification code | National standard | GB/T14946-94 A6 |
| 13 | Degree Code | National standard | GB6864 |
| 14 | Foreign language proficiency code | National standard | GB6865 |
| 15 | Code for the purpose of going abroad | National standard | GB/10301 |
| 16 | Source code for overseas funding | National standard | GB/T14946 A28 |
| 17 | Qualification route code | National standard | GB/T14949-94 A11 |
| 18 | Learning method code | National standard | GB/T14946-94 A25 |
| 19 | Learning form code | National standard | GB/T14946-94 A3 |
| 20 | List of Class Codes of School Units | National standard | GB/T14946-94 A26 |
| 21 | Education and training nature code | National standard | GB/T14946-94 A24 |
| 22 | Education and training result code | National standard | GB/T140946-94 A7 |
| 23 | Unit nature code | National standard |  |
| 24 | Cadres source code | National standard | GB/T14946-94 A15 |
| 25 | Appointment code | National standard | GB/T14946-94 A12 |
| 26 | Flow reason code | National standard | GB/T14946-94 A16 |
| 27 | Employment Form Code | National standard | GB/T14946-94 |
| 28 | Salary category code | National standard | GB/T14946-94 |
| 29 | Wage change reason code | National standard | GB/T14946-94 A32 |
| 30 | Current job status code | National standard | GB/T14946-94 A8 |
| 31 | Job reason code | National standard | GB/T14946-94 A5 |
| 32 | Code of Employment Mode | National standard | GB/T14946 A4 |
| 33 | Dismissal reason code | National standard | GB14946-94 A10 |
| 34 | Dismissal method code | National standard | GB/T14946-94 A9 |
| 35 | Job change category code | National standard | GB/T14946-94 A7 |
| 36 | Honorable rewards code | National standard | GB8563+GB8560+GB8562 |
| 37 | Reward and punishment reason code | National standard | GB/T14946-94 A23 |
| 38 | Reward and punishment category code | National standard | GB/T14946-94 A20 |
| 39 | Political outlook code | National standard | GB4762-84 |
| 40 | Code of abnormal political appearance | National standard | GB/T14946-94 A5 |
| 41 | Gender code | National standard | GB2261-80 |
| 42 | Marriage status code | National standard | GB4766 |
| 43 | Health code | National standard | GB4767-84 |
| 44 | Education code | National standard | GB4658 |
| 45 | Account code | National standard | GB/T14946-94 |
| 46 | Individual income tax payment standard code | National standard |  |
| 47 | Family Origin Code | National standard | GB4765 |
| 48 | My ingredient code | National standard | GB4764 |
| 49 | Personal identification code | National standard | GB/T14946 A1 |
| 50 | Kinship code | National standard | GB4761 |
| 51 | Hong Kong, Macao and Taiwan Overseas Chinese Code | National standard | GB/T14946 A2 |
| 52 | Worker technical grade code | National standard | GB/T14946-94 A13 |
| 53 | Military rank code | National standard | GB/T14946-94 A35 |
| 54 | School code | Recommended standards of the Ministry of Personnel |  |
| 55 | Professional code | Recommended standards of the Ministry of Personnel |  |
| 56 | SNOMEN (standard medical reference term) | International Standard |  |

**Table S2. Eight tasks which covered the main functional models of the patient app**

| Number | Tasks |
| --- | --- |
| Task 1 | To find and review a health report |
| Task 2 | To find and review action plans for diet, exercise, smoking, and medication management |
| Task 3 | To input health behavior information |
| Task 4 | To find and review a health behavior report |
| Task 5 | To find and review the health recommendations |
| Task 6 | To record blood pressure, blood sugar, blood lipids, or heart rate |
| Task 7 | To find and review a health educational article |
| Task 8 | To conduct a health consultation |

**Table S3.** **The task completeness levels**

| Number | Task completeness levels |
| --- | --- |
| 1 | No problem, easy to complete, no difficulty observed |
| 2 | A minor problem, having some difficulties or obstacles |
| 3 | A moderate problem, able to complete the task, but there are some problems during the process or have to adopt a cumbersome method to complete the task |
| 4 | A major problem, failed or gave up, unable to complete the task for some reasons |

**Table S4.** **The severity ranking of usability problem**

| Rankings | The severity ranking of usability problem |
| --- | --- |
| 0 | I don’t agree that this is a usability problem at all |
| 1 | Cosmetic problem only: need not to be fixed unless extra time is available |
| 2 | Minor usability problem: fixing this should be given low priority |
| 3 | Major usability problem: important to fix, so should be given high priority |
| 4 | Usability catastrophe: imperative to fix this before product can be released |

**Table S5. Basic Characteristics of patients enrolled in the first-step usability study**

|  | **Number** | **Percentage (%)** |
| --- | --- | --- |
| Gender |  |  |
| Male | 62 | 71.3 |
| Female | 25 | 28.7 |
| Age (Mean, SD) | 60 | 9.9 |
| Education level |  |  |
| Primary school | 10 | 11.7 |
| Middle school | 22 | 25.6 |
| High school or equivalent | 32 | 37.2 |
| College level or above | 22 | 25.6 |
| Occupation |  |  |
| Retired | 55 | 75.3 |
| Technician | 6 | 8.2 |
| Office clerk | 4 | 5.5 |
| Business managers | 2 | 2.7 |
| Workers | 2 | 2.7 |
| Farmer | 4 | 5.5 |
| Average monthly household income (US dollars) |  |  |
| < 257 | 3 | 3.5 |
| 257-429 | 19 | 22.4 |
| 429-1143 | 49 | 57.6 |
| ≥ 1143 | 14 | 16.5 |
| PCI | 25 | 28.4 |
| CABG | 2 | 2.3 |
| Medical history |  |  |
| Hypertension | 59 | 67.8 |
| Diabetes | 34 | 39.1 |
| Dyslipidemia | 60 | 69.0 |
| Cerebrovascular disease | 3 | 3.4 |
| Unhealthy behaviors |  |  |
| Unhealthy diet | 52 | 61.9 |
| Physical inactive | 36 | 42.9 |
| Current smoker | 28 | 33.3 |
| Medication non-adherent | 40 | 47.6 |

SD, standard deviation; PCI, percutaneous coronary intervention; CABG, coronary artery bypass grafting.

**Table S6. Identified usability problems and severity rankings**

| Tasks | Usability problem category | Usability problems | Severity Ranking |
| --- | --- | --- | --- |
| Task 1 | Category 4 | The font of the health report is too small | 3 |
|  | Category 1 | I don’t remember where the health report is | 1 |
|  | Category 4 | The font of the home page is too small | 2 |
| Task 2 | Category 4 | The font of the action plan is too small | 3 |
|  | Category 2 | The content of the action plan does not match to my eating habits | 2 |
| Task 3 | Category 2 | I don’t know how to record how much I ate | 2 |
|  | Category 3 | I don’t know how to choose the unit for a particular food when recording foods | 1 |
|  | Category 3 | When recording the diet, the slide gauge is hard to operate | 4 |
|  | Category 4 | The icon for diet is not obvious | 2 |
| Task 4 | Category 4 | I do not understand about the different colors in the medication health report | 1 |
| Task 5 | Category 5 | The user interface changes too fast | 2 |
| Task 6 | Category 5 | In the blood pressure modules, I did not know that the two circles of the icon can be moved | 2 |
|  | Category 5 | When recording blood pressure, the icon is not easy to operate | 4 |
|  | Category 5 | I don’t know the outside circle of the blood pressure icon is for the systolic blood pressure | 2 |
|  | Category 5 | The two circles of the blood pressure icon are too close | 4 |
| Task 7 | Category 7 | I thought the health educational articles are the health intervention messages | 2 |
| Task 8 | Category 6 | The response for the consultation is not timely | 4 |


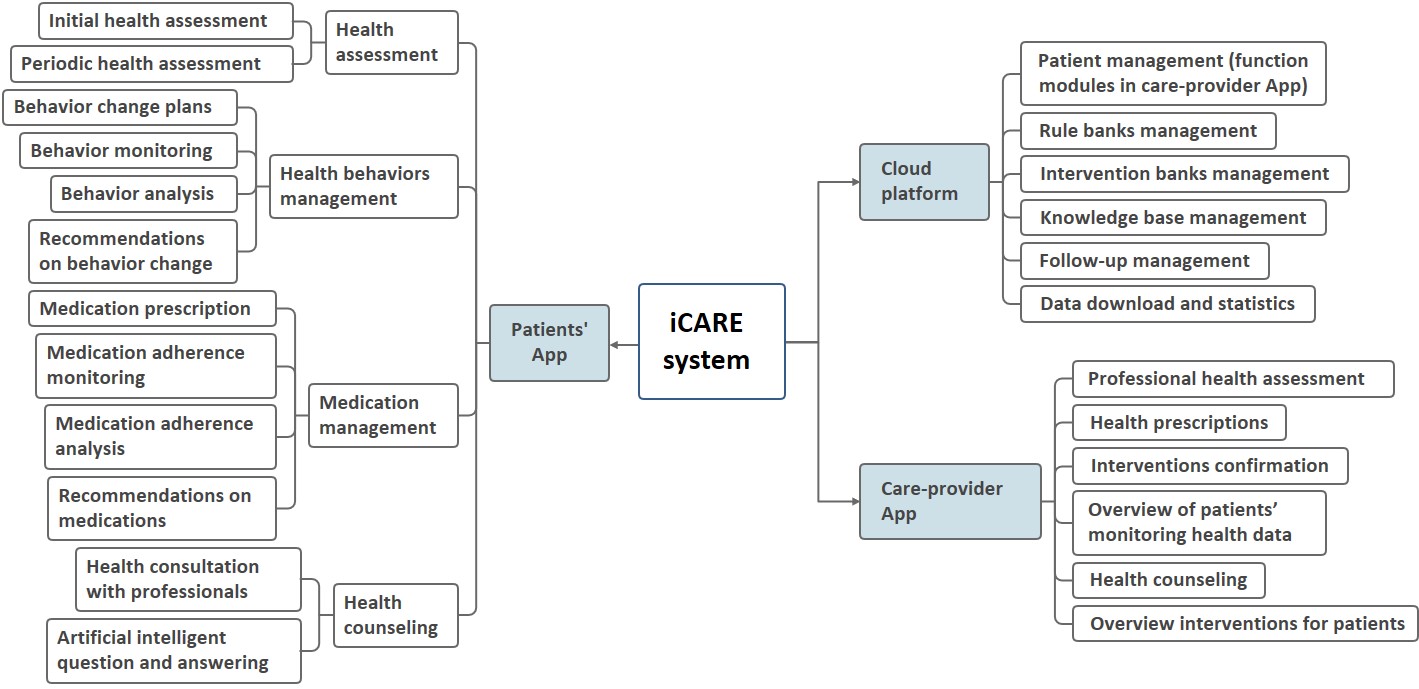


**Figure S1.** **The overall functional modules of the iCARE system**

**
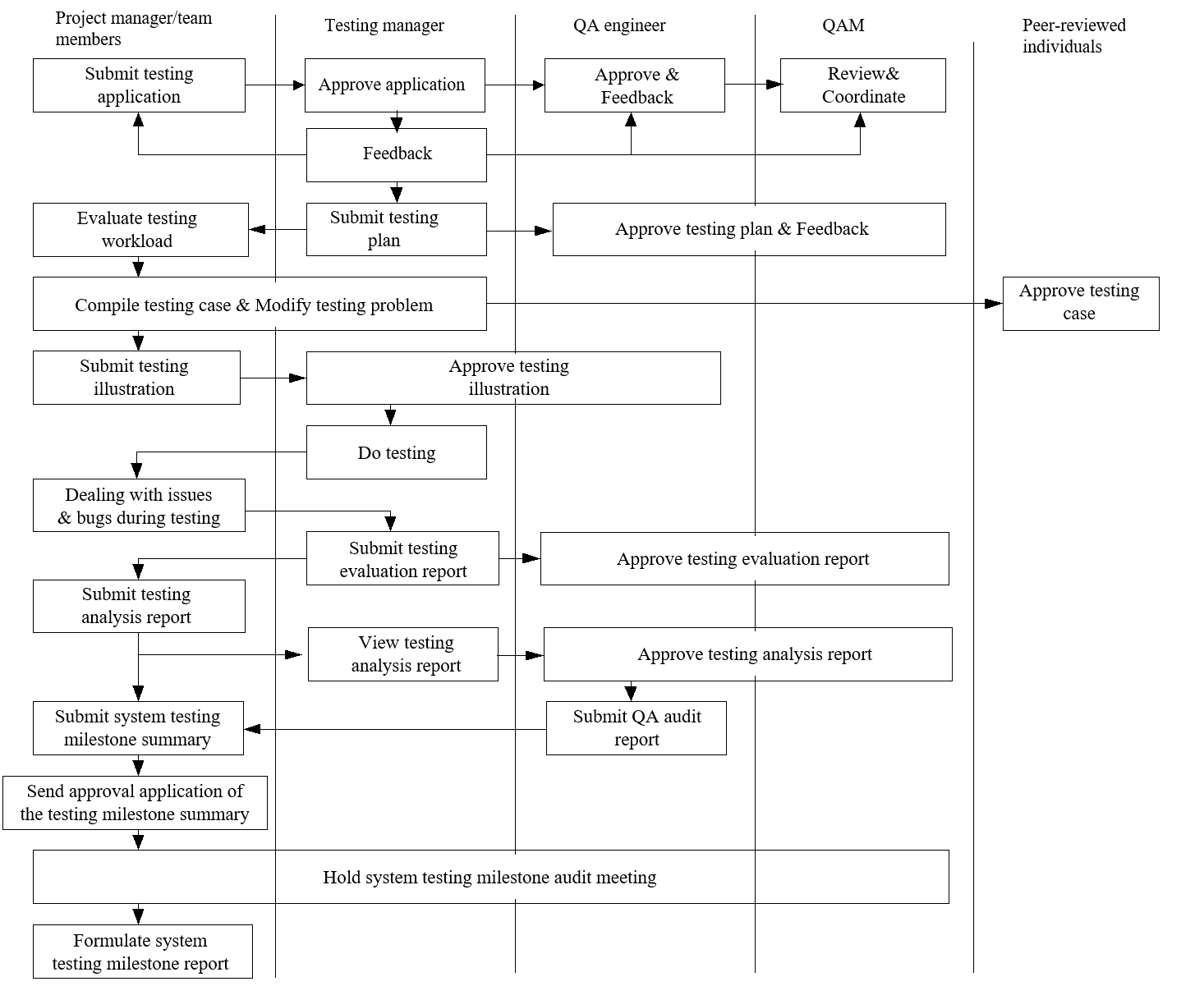
**

**Figure S2. The diagram on alpha version testing of the iCARE system**


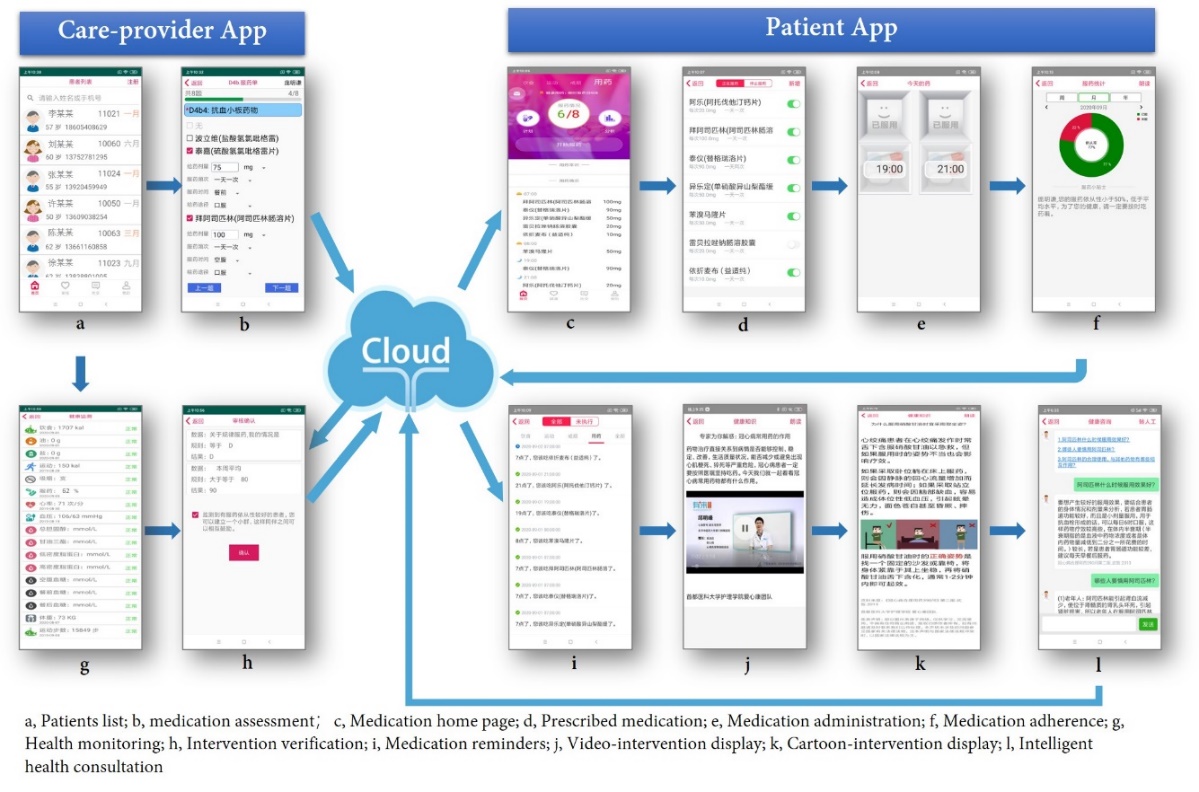


**Figure S3. The diagram of user interface for medication management**


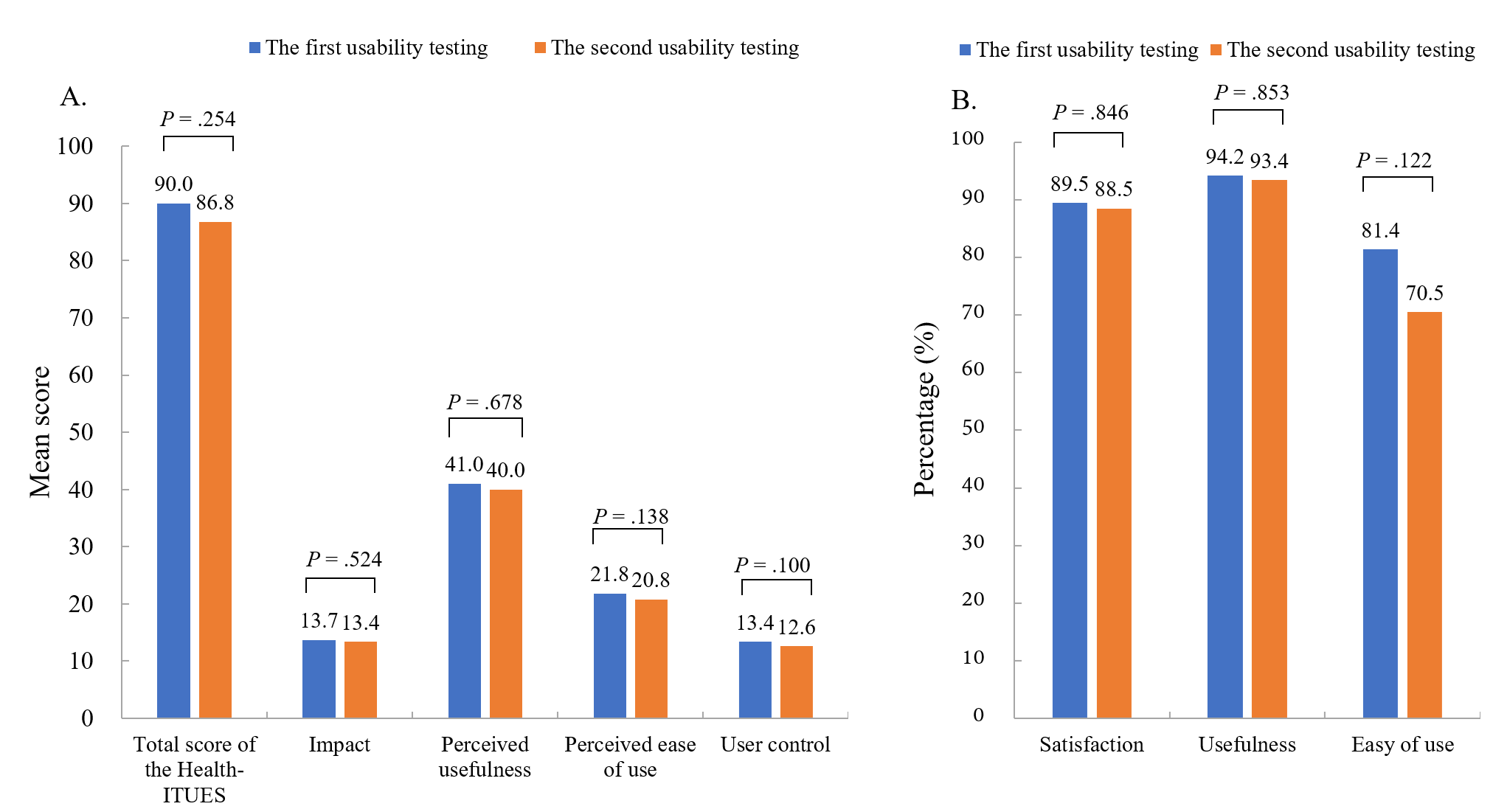


**Figure S4. Comparison of usability testing results in the first and second usability testing**

A. The Health-ITUES scores and the four dimensions of the Health-ITUES in the first and second usability testing. B. The percentage of satisfaction, usefulness, and ease to use in the first and second usability testing. Health-ITUES, the modified Health Information Technology Usability Survey.
